# A phylogenetically novel cyanobacterium most closely related to *Gloeobacter*

**DOI:** 10.1038/s41396-020-0668-5

**Published:** 2020-05-18

**Authors:** Christen L. Grettenberger, Dawn Y. Sumner, Kate Wall, C. Titus Brown, Jonathan A. Eisen, Tyler J. Mackey, Ian Hawes, Guillaume Jospin, Anne D. Jungblut

**Affiliations:** 1 University of California Davis, Department of Earth and Planetary Sciences, Davis, CA USA; 2 University of California Davis Genome Center, Davis, CA USA; 3 University of California Davis, Veterinary Medicine Population Health and Reproduction, Davis, CA USA; 4 Massachusetts Institute of Technology, Department of Earth, Atmospheric, and Planetary Sciences, Cambridge, MA USA; 5 University of Waikato, Tauranga, New Zealand; 6 The Natural History Museum, London, Life Sciences Department, Cromwell Road, London, SW7 5BD United Kingdom

**Keywords:** Bacterial genetics, Phylogenetics

## Abstract

Clues to the evolutionary steps producing innovations in oxygenic photosynthesis may be preserved in the genomes of organisms phylogenetically placed between non-photosynthetic Vampirovibrionia (formerly Melainabacteria) and the thylakoid-containing Cyanobacteria. However, only two species with published genomes are known to occupy this phylogenetic space, both within the genus *Gloeobacter*. Here, we describe nearly complete, metagenome-assembled genomes (MAGs) of an uncultured organism phylogenetically placed near *Gloeobacter*, for which we propose the name Candidatus *Aurora vandensis* {Au’ro.ra. L. fem. n. aurora, the goddess of the dawn in Roman mythology; van.de’nsis. N.L. fem. adj. vandensis of Lake Vanda, Antarctica}. The MAG of *A. vandensis* contains homologs of most genes necessary for oxygenic photosynthesis including key reaction center proteins. Many accessory subunits associated with the photosystems in other species either are missing from the MAG or are poorly conserved. The MAG also lacks homologs of genes associated with the pigments phycocyanoerethrin, phycoeretherin and several structural parts of the phycobilisome. Additional characterization of this organism is expected to inform models of the evolution of oxygenic photosynthesis.

## Introduction

Around 2.4 billion years ago, Earth’s surface environments changed dramatically. Atmospheric oxygen rose from <10^−5^ times present atmospheric level (PAL) to >1% PAL [1–4**]**. This Great Oxygenation Event (GOE) permanently changed Earth’s surface geochemistry, fundamentally reshaped the cycling of key elements [5] and altered the evolutionary path of life by allowing widespread oxygen respiration [6, 7]. The GOE was enabled by photosynthetic oxygen production by Cyanobacteria, making the evolution of oxygenic photosynthesis one of the most important innovations in Earth’s history [4, 8]. However, the evolutionary processes leading to oxygenic photosynthesis are poorly constrained [9–13]. In one hypothesis, Cyanobacteria acquired photosynthetic genes for both photosystems I and II (PSI and PSII, respectively) via horizontal gene transfer and then combined and refined them to form the photosystems that drive oxygenic photosynthesis in Cyanobacteria [14, 15]. In another hypothesis, the common ancestor of all phototrophic bacteria contained the genes necessary for photosynthesis, which diversified through time and were selectively lost in non-phototrophic portions of those lineages [16–20]. In either scenario, the gene content of photosystems in Cyanobacteria can help elucidate the evolutionary processes leading to oxygenic photosynthesis.

Researchers have attempted to extract evolutionary information by studying the photosynthetic genus *Gloeobacter*, which is an outgroup to all other Cyanobacteria [21–24]. In these studies, traits absent from *Gloeobacter* species but common in other Cyanobacteria are interpreted to have evolved after the divergence of *Gloeobacter*. For example, the *Gloeobacter* do not contain thylakoid membranes, which host the photosynthesis enzymes in other Cyanobacteria [25, 26]. In *Gloeobacter* species, photosynthesis and respiration occur in the cytoplasmic membrane rather than in the thylakoid membrane [27]. *Gloeobacter* spp. also contain structurally simple phycobilisomes, the protein complex responsible for absorbing photons and transferring energy to the PSII reaction center. The six rods of the *Gloeobacter* phycobilisome form a single bundle whereas many phycobilisomes are hemidiscoidal in many other Cyanobacteria [28]. However, pycobilisome structure can vary within the Cyanobacteria. For example, Acaryochloria marina contains both rod and hemidiscoidal structures [29, 30] Additionally, *Gloeobacter* spp. lack several PSII proteins, including PsbY, PsbZ and Psb27, and others are poorly conserved, including PsbO, PsbU, and PsbV [31]. The absence of the thylakoid membrane, differences in light harvesting, and missing photosynthesis proteins have been used by some researchers to constrain models for the evolution of oxygenic photosynthesis as well as the ecology and photochemistry of ancestral Cyanobacteria [23, 24, 32].

The Vampirovibrionia are a sister group to all Cyanobacteria, including *Gloeobacter* [10, 11, 33], and researchers have also interrogated their genomes for insights into the evolution of oxygenic photosynthesis [10–12, 33, 34]. No characterized Vampirovibrionia have genetic content that indicates a potential for phototrophy [10, 11, 33]. The absence of known phototrophic Vampirovibrionia could be due to: (1) incomplete characterization of the clade, which may contain some as yet unidentified phototrophs; (2) the genes necessary for photosynthesis were present in a common ancestor of Vampirovibrionia and Cyanobacteria and then lost in Vampirovibrionia and related lineages [35] or (3) oxygenic photosynthesis evolved after the divergence of Vampirovibrionia and Cyanobacteria [10–12, 33].

An undescribed group of organisms, known only from 16S rRNA gene surveys, is phylogenetically placed near the *Gloeobacter* and may provide insights into innovations in oxygenic photosynthesis. We recovered two nearly compete metagenome-assembled genomes (MAGs) of this taxon from microbial mats in Lake Vanda, McMurdo Dry Valleys, Antarctica, and we propose to name the taxon Candidatus *Aurora vandensis*.

Candidatus *Aurora* (Au’ro.ra. L. fem. n. aurora, the goddess of the dawn in Roman mythology, referring to the organism preference for inhabiting high latitude locations with low light).

Candidatus *Aurora vandensis* (van.de’nsis. N.L. fem. adj. vandensis of Lake Vanda, Antarctica, referring to the place where the organism was found).

## Methods

### Site description

Lake Vanda is a perennially ice-covered lake located within Wright Valley, McMurdo Dry Valleys, Antarctica. Lake Vanda has a perennial ice cover of 3.5–4.0 m. The ice cover transmits 15–20% of incident photosynthetically active radiation [36]. Wavelengths shorter than 550 nm dominate the light spectrum because ice transmits little red light and water is particularly transparent to blue-green light [37]. Nutrient concentrations are low, and therefore there is little biomass in the water column [38]. However, benthic mats are abundant [39], covering the lake bottom from the base of the ice to >50 m [40]. The microbial mats contain abundant 0.1–30 cm tall pinnacles [41] that incorporate annual mud laminae. Mat surfaces have brown-purple coloration due to trapped sediment and pigments. The underlying layers are characterized by green and purple pigmentation. The inner sections of large pinnacles are comprised of beige decomposing biomass. The dominant cyanobacterial genera based on morphological and 16S rRNA gene surveys are trichome-forming *Leptolyngbya, Pseudanabaena, Wilmottia, Phormidium, Oscillatoria* and some unicellular morphotypes [41, 42]. The microbial mats also contain diverse algae, especially diatoms, and other bacteria and archaea [39, 43]. Incident irradiance penetrates millimeters into the mats, and samples from the interior of the mat analyzed here were exposed to low irradiance (<1 µmol photons m^−2^ s^−1^) in their natural environment [37].

### Sampling and DNA extraction

To obtain samples, SCUBA divers collected intact benthic microbial mats from 9 and 19 m lake depth and brought them to the surface in sterilized plastic containers. Pinnacles were either kept intact as bulk samples or dissected in the field based on color using sterile technique. Subsamples were placed in Zymo Xpedition buffer (Zymo Research, Irvine, CA), and cells were lysed via bead beating in the field. The stabilized samples were then frozen on dry ice and maintained frozen in the field. Samples were transported at −20 °C to UC Davis. DNA was extracted at UC Davis using the QuickDNA Fecal/Soil Microbe kit using the manufacturer’s instructions (Zymo Research, Irvine, CA, USA). The extracted DNAs were quantified using Qubit (Life Technologies) and were concentrated via evaporation until the concentration was ≥ 10 ng/uL. The one green mat and one purple subsample used for this project were sequenced at the US Department of Energy Joint Genome Institute (JGI).

### DNA sequencing

The JGI generated sequence data using Illumina technology and reads were quality controlled using their in-house pipeline. Briefly, an Illumina library was constructed and sequenced 2 ×151 bp using the Illumina HiSeq-2500 1TB platform. BBDuk (version 37.36) was used to remove common contaminants (removehuman=t, removedog=t, removecat=t, removemout=t, and removemicrobes=t), trim reads that contained adapter sequence and bases to the right of bases where the quality score drops to 0. BBDuk was also used to remove reads that contained 4 or more ‘N’ bases, had an average quality score across the read less than 3 or had a minimum length ≤ 51 bp or 33% of the full read length. Reads mapped to masked human, cat, dog and mouse references at 93% identity were removed. Reads aligned to common microbial contaminants were also removed.

### Bioinformatic analysis

Quality controlled, filtered raw data were retrieved from IMG Gold (JGI Gold ID GP0191362 and Gp0191371). Metagenomes were individually assembled using MEGAHIT 1.0.6 [44] using a minimum contig length of 500 bp and the paired end setting. Reads were mapped back to the assembly using Bowtie2 1.2.2 [45]. A depth file was created using jgi_summarize_bam_contig_depths and the assemblies were binned using MetaBAT with a minimum contig length of 2500 bp [46]. CheckM 1.0.7 assessed the quality of the bins [47], and bins of interest were identified based on phylogenetic placement. Average nucleotide identity (ANI) was calculated using the OrthoANI algorithm [48]. Protein-coding regions were identified by prodigal V2.6.3 [49] within CheckM. GhostKOALA and Prokka 1.12 were used to annotate translated protein sequences [50, 51]. Nucleotide and translated nucleotide sequences from the MAGs are available on OSF.io [52]. The whole-genome shotgun project has been deposited at Genbank under the accessions JAAXLU000000000 and JAAXLT000000000. The versions described in this paper are versions JAAXLU010000000 and JAAXLT010000000.

When homologs of genes from the KEGG photosynthesis module were not present in the bin, they were searched for in assembled, unbinned data by performing a BLASTX search with an E-value cutoff of 1E-5. BLASTP was used to find the best hit for the retrieved sequences and to exclude those that were not the target gene. Any unbinned sequences phylogenetically similar to *A. vandensis* were identified as belonging to the MAGs based on their position in a phylogenetic gene tree constructed using the methodology described below.

### Phylogenetic inference

Aligned, nearly full-length 16S rRNA gene sequences (~1000–1500 bp) of reference Cyanobacteria were collected from the Silva database (v123; [53, 54]). We recovered a single 16S rRNA gene sequence from the green mat MAG and this sequence was added to the overall alignment using MAFFT [54]. A maximum-likelihood tree was constructed in RAxML-HPC2 on XSEDE [55] in the CIPRES Science Gateway [56]. The tree was rooted and visualized in the interactive tree of life [57] (Supplemental File 1). A maximum-likelihood tree based on 16S rRNA gene sequences were separately constructed in MEGA7 [58]. For this tree, sequences were aligned with Muscle [59] and a maximum-likelihood tree was constructed using the Tamura-Nei model [60] with uniform rates among sites and complete deletion and 100 bootstrap replicates. The maximum-likelihood heuristic model was nearest-neighbor interchange (Supplemental File 2).

A maximum-likelihood tree was constructed using translated nucleotide sequences of single-copy marker genes including IF3 C-terminal and L2 proteins (Supplemental Files 3, 4). Sequences were aligned in Muscle [59] in the CIPRES Science Gateway [56] using default parameters. A maximum-likelihood tree was constructed in RAxML-HPC2 on XSEDE [55] in the CIPRES Science Gateway [56] as described above.

The D1 sequence from the green subsample was added to a previously generated alignment in MAFFT [18, 54]. A maximum-likelihood tree was constructed in RAxML-HPC2 on XSEDE [55] in the CIPRES Science Gateway [56] as described above (Supplemental File 5).

A concatenated, single-copy marker gene tree was constructed from the MAGs, published Vampirovibrionia and Sericytochromatia genomes and one genome for each genus of NAG Cyanobacteria available from the Integrated Microbial Genomes and Microbiomes (IMG) database using a custom wrapper script leveraging Phylosift’s search and align functions [61–63]. The alignments were generated by using Phylosift’s search and align functions using lastal and hmm align to identify 37 mostly single-copy marker genes as well as 16S sequences (Table S1). A maximum-likelihood tree was constructed as described above and trees were rooted and visualized in the interactive tree of life [57] (Supplemental File 6). Alignment files are available on osf.io [52].

## Results

Assembled metagenomes contained 313–1306 Mbp in 228,837–861,358 contigs with a mean sequence length of 1301–1669 bp. 49.6 and 53.3% of unassembled reads mapped back to the assembly for the green and purple samples, respectively. We recovered two bins of a taxon most closely related to *Gloeobacter*, one from each sample. Hereafter, we will refer to the taxon represented by the bins as a MAG. The bins were 3.07 and 2.96 Mbp in total length, had a GC content of 55.4% and 55.3%, and contained 3025 and 3123 protein-coding sequences respectively. Bins were 90.1 and 93.2% complete with 1.7 and 0.85% contamination based on marker gene analysis in CheckM. GhostKOALA annotated 41.1 and 41.7% of the predicted protein-coding sequences. Marker gene sequences and key photosynthetic gene sequences from the bins were identical or nearly identical and the genomes were 99.96% similar based on ANI.

**Fig. 1:**
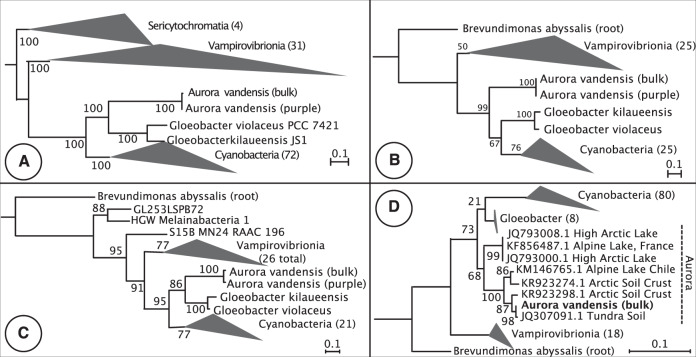
Phylogeny of A. vandensis. **a** Genome phylogeny based on the concatenation of 37 single-copy marker genes showing *A. vandensis* as a sister group to the *Gloeobacter;* **b** 16S rRNA gene phylogeny showing the genus *Aurora* as a sister group to all other Cyanobacteria; **c** Ribosomal protein L2 phylogeny with *A. vandensis* sister to the *Gloeobacter;* and **d** IF3 C terminal phylogeny showing *A. vandensis* as sister to the *Gloeobacter*.

The MAG is most similar to *G. violaceous* with which it has 69.9% ANI across the genome and *G. kilauensis* with which it has 69.8% ANI across the genome. The Phylosift concatenated marker gene tree placed the MAG as a sister group to the *Gloeobacter* (Fig. 1a). The individual marker gene trees differed in their topologies. For example, *A. vandensis* is a sister group to all other photosynthetic Cyanobacteria in the IF3 C terminal phylogeny and is a sister group to the *Gloeobacter* in the ribosomal protein L2 phylogeny (Fig. 1b, c). Phylogenetic trees based on 16S rRNA gene sequences varied and placed the MAG either as a sister group to all other Cyanobacteria (Fig. 1d) or as a sister to the *Gloeobacter* (Figure S1) dependent on the program used for tree building and the16S rRNA gene taxa included in the analyses. The 16S rRNA gene from the MAG was >99% similar to clones from moss pillars in an Antarctic lake (AB630682) and tundra soil (JG307085) and was 91.2% similar to *G. violaceous* strain PCC 7421 and 90.8% similar to *G. kilaueensis* (NR_074282; Fig. 1d).

**Fig. 2:**
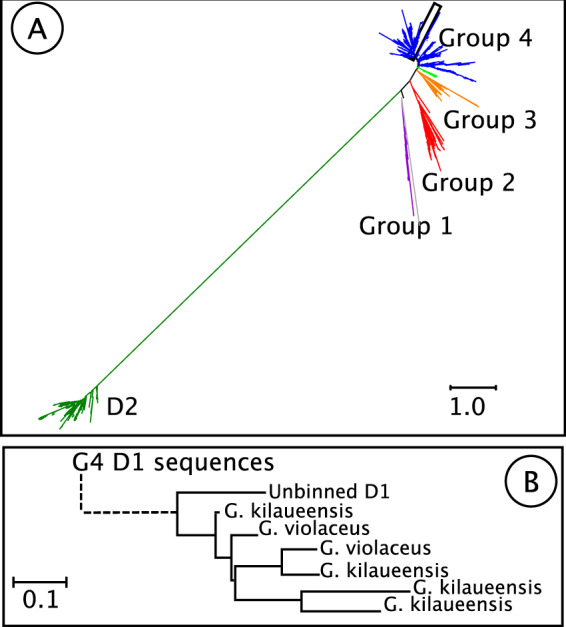
Phylogenetic position of D1 protein located in the green mat assembly. **a** D1 and D2 phylogeny showing D1 groups as defined by [18] Location of putative *A. vandensis* D1 highlighted by black box. **b** Region of tree highlighted by black box in A showing the putative *A. vandensis* sequence and its closest relatives.

Based on KEGG annotations, the MAG contained homologs of all the genes necessary for carbon fixation via the Calvin Cycle. It also contained many of the genes necessary for glycolysis via the Embden–Meyerhof–Parnas pathway (EMP; missing *pfkABC*) and citrate cycle. The MAG contains no genes associated with nitrogen fixation. The MAG contained homologs of chlorophyll biosynthesis genes *chlB, chlL,chlN, chlD*, and *chlM* but did not contain homologs of *chlH, chlI, chlG, or chlP*. The MAG contained homologs of many genes associated with oxygenic photosynthesis, but *psbM*, *psbZ*, *psbY*, *psb27*, and *psbU* from photosystem II (PSII) were not found. Similarly, homologs of *psbA* were absent from the bin, but a BLASTX search of assembled, unbinned data located a *psbA* sequence that is a sister to the Gloeobacter D1 group 4 sequence (Fig. 2). This sequence is on a contig that was too short to be binned. PSII genes *psbJ,psbP*, *psbO*, and *psbV* were annotated in the MAG but were dissimilar to those found in other Cyanobacteria (Table S2). The MAG lacked homologs of genes encoding phycobilisome proteins ApcD, ApcF, CpcD, RpcG, and CpcG, and any genes associated with phycoerythrocyanin (PEC) or phycoerythrin (PE) (Table S2). The PSI genes *psaI*, *psaJ*, *psaK*, and *psaX*, and the photosynthetic electron transport gene *petJ* (cytochrome c6) were also absent. Homologs of chlorophyll biosynthesis genes chlB, chlN, chlM, and chlN were also present in the MAG. For each missing photosynthesis gene, no homologs were found in the assembled, unbinned data that had similar phylogenetic placements to other genes in the MAG, except *psbA* as described above.

## Discussion

### Genus and species description

We propose that our MAG is the first genome within a new genus. It has a 66.8% average nucleotide identity (ANI) and approximately 91% similarity of its 16S rRNA gene with *G. violaceous* strain PCC 7421 and *G. kilauensis*, the most similar taxa. On average, genera contain taxa that are 96.5% similar based on 16S rRNA genes. Therefore, we propose the creation of a new genus, *Aurora*, which includes our MAG, *Aurora vandensis*, and numerous representatives in 16S rRNA gene sequence databases, nearly all from polar or alpine environments (Fig. 1). The candidate genus is named after Aurora, the goddess of the dawn, to reflect its early divergence from other Cyanobacteria and its presence in low light environments. Aurora also refers to the northern and southern lights, aurora borealis and aurora australis, mirroring *Aurora*’s apparent preference for high latitude locations. The species, *A. vandensis*, is named after Lake Vanda where the samples originated. Lake Vanda was named after a sled dog used in the British North Greenland Expedition [64].

The phylogenetic placement of *A. vandensis* varies based on the genes or proteins used to construct the phylogeny and the taxa included in the analysis (e.g., Fig. 1). In most analyses it was placed as a sister to the *Gloeobacter*, but phylogenies of the IC3 C terminal and a 16S rRNA gene phylogeny placed it also as a sister group to all other cyanobacterial lineages, e.g., non-*Aurora*, non-*Gloeobacter* Cyanobacteria (hereafter NAG Cyanobacteria). Additionally, *A. vandensis* and *Gloeobacter* are located in a portion of the phylogenetic tree that is (a) at the end of a long branch and (b) does not contain many sequenced genomes. Therefore, we cannot be confident in the phylogenetic placement of *A. vandensis*. Additional genomes may help to better resolve *Aurora*’s family-level classification within the Cyanobacteria.

To date, *Aurora* is composed of taxa from high altitude or high latitude regions including Arctic microbial mats [65], Patagonian Andes [66], Nunavut, Canada [67], the French Alps [68], and perennially ice-covered lakes in Antarctica [69] and the current study (Fig. 1d), with a single taxon from modern stromatolites in Tasmania [70]. Based on this geographic distribution, *Aurora* may be a cold-adapted clade [65, 71].

### Metabolic characterization of the uncultured *Aurora* genome

Based on its genome content, *A. vandensis* is predicted to be capable of both carbon fixation via the Calvin Cycle and glycolysis via the EMP. Many Cyanobacteria use the EMP pathway to ferment glycogen under dark conditions [72, 73], and *A. vandensis* may do so in Lake Vanda during the four months of darkness over the Antarctic winter.

*A. vandensis* is likely capable of performing oxygenic photosynthesis, but the MAG does not contain homologs to proteins that act as accessory subunits present in most Cyanobacterial photosystems. The lack of these genes may result from the incompleteness of the MAG or because they are actually missing from the *A. vandensis* genome. Here, we provide an analysis of the potential metabolic consequences if these proteins are actually absent in the *A. vandensis* genome rather than missing due to bin completeness.

### Thylakoid membrane

In NAG Cyanobacteria, most photosynthetic processes occur in the thylakoid membrane, but this membrane is absent in *Gloeobacter* species, and photosynthesis and respiration occur in the cellular membrane [25, 26]. Few genes are known to be associated with the construction of the thylakoid membrane in Cyanobacteria. The VIPP1 gene is important [74] or essential to building a thylakoid membrane in organisms capable of oxygenic photosynthesis and is absent from *Gleobacter* [75]. It is also not present in the MAGs. Therefore A. vandensis may not contain thylakoid membranes.

### Light harvesting

*A. vandensis* is predicted to be capable of collecting light energy for use in photosynthesis despite lacking homologs of several phycobiliproteins used for light-harvesting proteins in some other Cyanobacteria. Phycobilisomes harvest photons for use in PSII. These structures contain stacks of pigment proteins (biliproteins) connected by linker proteins, which are anchored into the thylakoid membrane in NAG Cyanobacteria or the cell membrane in the *Gloeobacter*. The pigments in many phycobilisomes include a core of allophycocyanin which best captures photons at ~650 nm, surrounded by rods of phycocyanin (~620 nm), phycoerythrin (maxima between 495–560 nm) and phycoerythrocyanin (575 nm). The *A. vandensis* MAG lacks homologs for genes associated with phycoerythrin and phycoerythrocyanin. Therefore, *A. vandensis* may have either lost those genes or they may have evolved after the divergence of *A. vandensis* from other Cyanobacteria. *Gloeobacter* spp. also lack genes for phycoerythrocyanin, so if the last common ancestor for *Aurora* and *Gloeobacter* had phycoerythrocyanin genes, they were lost in both lineages. In contrast, phycoerythrin-associated genes are present in *Gloeobacter* spp. so these may have been present in a common ancestor of *A. vandensis* and *Gloeobacter* but lost in *A. vandensis*. Cyanobacteria can lose biliproteins in response to their light environment. For example, *Leptolyngbya* sp. BC1307, isolated from the McMurdo Dry Valley Lake Hoare, Antarctica, lacks phycoerythrin, which may prevent it from being photoinhibited [76]. Alternately, the lack of these proteins may be an adaptive strategy that allows it to reduce the number of resources directed to the phycobilisome as has been proposed for other polar phototrophs [76–79].

Biliprotein linker proteins transfer energy from the biliproteins to the reaction center [31, 80] and neither *A. vandensis* nor *Gloeobacter* encode homologs of some linker proteins that are present in other Cyanobacteria. Homologs for the phycobilisome rod-core linker protein for PEC, *cpcG*, are absent in *Gloeobacter* (and *A. vandensis)*. Instead, *Gloeobacter* uses *cpcJ* (Glr2806), which connects phycocyanin and allophycocyanin, and *cpeG* (Glr1268*)*, which connects phycocyanin and PE. The *A. vandensis* MAG also lacks the phycoerythrocyanin linker proteins. In addition, the sequences that may be homologous to *cpc*J and *cpe*G, in the *A. vandensis* MAG are not known to serve similar functions. The linker proteins that are absent from the *A. vandensis* MAG are not essential to oxygenic photosynthesis. Knockouts of these genes in other Cyanobacteria do not inhibit photosynthesis, but mutants have lower energy transfer to the reaction center than wildtype strains [81].

Overall, *A. vandensis* is predicted to capture irradiance for photosynthesis using allophycocyanin and phycocyanin and to transfer that energy to PSII via the associated linker proteins.

### Photosystem II reaction center

**Fig. 3:**
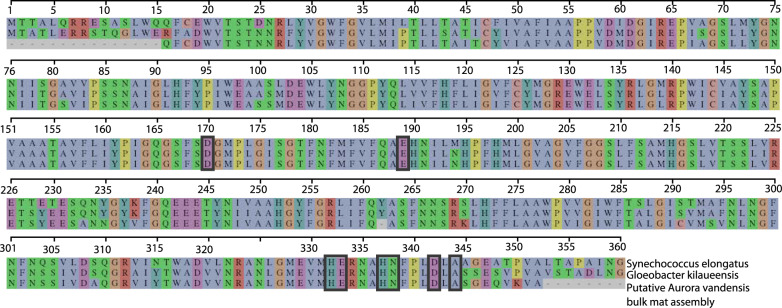
Alignment of D1 protein sequences from *Synechococcus elongatus* and *Gloeobacter kilaueensis* and the translated D1 sequence from the green mat assembly.

To perform oxygenic photosynthesis, *A. vandensis* is expected to need a functional PSII reaction center, including the D1and D2 proteins and other proteins structuring the oxygen-evolving center (OEC). The *A. vandensis* MAG did not contain *psbA*, which encodes the D1 protein. However, we located a D1 sequence from each of the assemblies that is phylogenetically located at the same place as *A. vandensis* (Fig. 2), is 91% similar to that of *G. violaceus* (WP_023172020), and is located on a contig that is too short to be binned using our methods (<2500 bp). Based on these attributes, we hypothesize that these *psbA* sequences belong to the *A. vandensis* MAG. The translated *psbA* sequence produces a D1 protein within Group 4, the group that is functional for oxygen production and present in all known photosynthetic Cyanobacteria [18]. It contains all of the previously described amino acid ligands necessary to support the Mn_4_CaO_5_ cluster in the OEC (Asp170, Glu189, His332, Glu333, His 337, Asp342, and Ala344) [82, 83] Fig. 3). Thus, this D1 protein is likely fully functional.

Most other critical PSII reaction center subunits have homologs in the *A. vandensis* MAG (Table S2), which suggests it is capable of oxygenic photosynthesis. However, the MAG lacks homologs to a number of genes involved in supporting the PSII reaction center (Table S2). The minimum suite of genes necessary for a functional PSII is not known. *Gloeobacter* spp. perform oxygenic photosynthesis without *psbY*, *psbZ*, and *psb27* [31] which are also absent from the *A. vandensis* MAG, suggesting that they are not essential. Other PSII homologs that are absent from the *A. vandensis* MAG (*psbM* and *psbU*) or are poorly conserved (*psbJ* and *psbV*) have been individually knocked out in NAG Cyanobacteria. Mutants lacking *psbM*, which helps stabilize the PSII D1/D2 dimer, still form functional D1/D2 dimers [84]. Those lacking the stabilizing *psbU* or *psbV* still perform oxygenic photosynthesis, but they have decreased energy transfer between the allophycocyanin phycobilisome and PSII [85], are highly susceptible to photoinhibition, have decreased light utilization under low-light conditions, and have lowered oxygen evolution and electron donation rates than the wildtype [86–88]. Mutants lacking *psbJ* have less stable D1/D2 dimers but still photosynthesize with lower rates of oxygen production than the wildtype [89]. Finally, the gene encoding PsbO is poorly conserved in *A. vandensis* relative to *Gloeobacter* (46%) and NAG Cyanobacteria (≤36%), compared with ≥55% similarity among NAG Cyanobacteria. Despite this, PsbO in *A. vandensis* contains all the features previously identified as necessary to interact with the D1, D2, CP43 and C47 subunits [90]. Therefore, the *A. vandensis* PsbO subunit likely helps stabilize the Mn_4_CaO_5_ cluster and support the OEC as it does in other Cyanobacteria.

PsbO, PsbU, PsbV, a region of the D1, and other extrinsic proteins help control the concentration of Cl^−^, Ca^2+,^ and H^+^ and create an environment that is amenable to water oxidation [84, 91–93]. Although PsbU is missing in the *A. vandensis* MAG, other PSII proteins conserve residues that have been identified as important for Cl^−^ and Ca^2+^ regulation. For example, the D1 chloride ligand site Asn338 is conserved in the translated *psbA* that we attribute to *A. vandensis* (Fig. 3). Similarly, the translated *psbO* contains Glu54, Glu114, and His231 residues that bind with Ca^2+^ [94], suggesting that *A. vandensis* is capable of some Cl^−^ and Ca^2+^ regulation.

Individually, each of the missing or poorly conserved PSII reaction center genes reduce energy transfer, growth rate, or oxygen evolution of oxygenic photosynthesis in mutated NAG Cyanobacteria. If the genes missing in the *A. vandensis* MAG are missing in the organism, this taxon may have a less stable OEC and lower oxygen evolution potential than other Cyanobacteria. However, none of the missing genes are individually necessary to perform oxygenic photosynthesis, suggesting that, if *A. vandensis* is missing all of the genes absent from the MAG, it could still be capable of oxygenic photosynthesis.

### Cytochrome b6f

Once through PSII, the electrons pass through the cytochrome b6f complex, which is composed of eight subunits in NAG Cyanobacteria. The *A. vandensis* MAG contains homologs of genes encoding six of these subunits, including PetA, PetB, PetC, PetD, PetM and PetG. However, the MAG is missing *petL* and *petN*. *Gloeobacter* is also missing *petL*, and a *Synechocystis* mutant was able to grow photoautotrophically without *petL*, although it produced oxygen at a reduced rate compared to the wildtype [95]. Therefore, an absence of *petL* should not inhibit the functionality of cytochrome b6f. The effect of an absence of *petN* is less clear. Deletion of *petN* prevented plants from photosynthesizing [96, 97]. These results have been interpreted to mean that *petN* is necessary for photosynthesis in both plants and Cyanobacteria [95, 98], but attempts to delete *petN* in Cyanobacteria have been unsuccessful [95]. Therefore, it is not possible to determine what effect its absence may have on electron transport if it is not present in *A. vandensis*. Overall, even if these genes are absent from *A. vandensis*, oxygenic photosynthesis and aerobic respiration should not be inhibited.

Cytochrome b6f passes electrons to either plastocyanin or cytochrome c6. *Aurora vandensis* contains homologs of genes necessary to produce plastocyanin, but the MAG lacks homologs of *petJ*, which codes for cytochrome c6. *petJ* knockout Cyanobacteria grow at the same rate as wildtype [99]. Therefore, *A. vandensis* would not be affected by the lack of cytochrome c6 even if *petJ* is absent from the organism because it could use plastocyanin as the final electron carrier delivering electrons to PSI.

### Photosystem I reaction center

The genetic makeup of PSI in *A. vandensis* MAG is similar to that in *Gloeobacter* genomes. Both contain the main subunits for PSI, but lack homologs of several genes including *psaI*, *psaJ*, and *psaK* that are present in NAG Cyanobacteria. However, the *A. vandensis* MAG also lacks homologs of *psaM*, which is present in *Gloeobacter*. PsaM is involved in forming stable PSI trimers, and Cyanobacteria mutants lacking this gene perform photosynthesis and grow at a normal rate [100]. The *A. vandensis* MAG also contains the genes encoding for a homolog of PsaZ, a protein only found in *Gloeobacter* species. This protein may stabilize the PSI structure in the absence of PsaI, PsaJ, and PsaK [32]. Therefore, PSI in *A. vandensis* likely functions similarly to PSI in *Gloeobacter*, even if *psaM* is absent from the organism.

### Photoprotection

Cyanobacteria can experience photoinhibition under high light conditions when photon absorption outstrips the ability to dissipate electrons through photochemical pathways, and reactive oxygen species accumulate at the PSII reaction center. These reactive species damage photosynthetic machinery, especially the D1 protein, which then requires reassembly [101]. Cyanobacteria protect themselves from photoinhibition in two key ways. First, they use orange carotenoid proteins (OCP) as receptors to reduce the amount of energy transferred from the phycobilisome to PSII and PSI [101]. The *A. vandensis* genome contains homologs of a gene coding for a protein 68% similar to the OCP in *G. violaceous*. Because the OCP interacts directly with the phycobilisome [101] the sequence differences may reflect structural differences in the phycobilisomes of *A. vandensis* and *G. violaceous*. Alternately, the OCP in *A. vandensis* may be belong to recently described paralogs of OCPs called helical carotenoid proteins [102–104]. Some of these proteins are capable of antennae or singlet oxygen quenching activity, but the function of others is unknown [102].

Cyanobacteria also protect themselves from photoinhibition using high light-inducible proteins (HLIP) to dissipate energy. *A. vandensis* contains homologs of genes for three proteins that are 69–85% similar to HLIP in *G. violaceous*. We hypothesize that these genes act as HLIP and protect *A. vandensis* against photoinhibition.

### Importance of *Aurora vandensis*

*A. vandensis*, *G. violaceous* and *G. kilauensis* are the only species identified to date occupying the phylogenetic space between the NAG Cyanobacteria and the Vampirovibrionia. The addition of the genus *Aurora* to this portion of the phylogenetic tree will provide key insights into evolutionary events associated with the divergence of two lineages with very different ecological distributions and dominant metabolisms, e.g., the NAG Cyanobacteria, which are ubiquitous in illuminated environments and which are nearly universally capable of oxygenic photosynthesis, and the Vampirovibrionia, none of which are known to be capable of phototrophy [10–12, 33].

## Supplementary information

Supplemental Captions

Figure S1.

Table S1.

Table S2.

Supplemental File 1

Supplemental File 2.

Supplemental File 3.

Supplemental File 4.

Supplemental File 5.

Supplemental File 6.
