# Supplementary material for: A phylogenetically novel cyanobacterium most closely related to Gloeobacter
Source: ISME J. 2020 May 18;14(8):2142–52. doi: 10.1038/s41396-020-0668-5 (PMC7368068; doi:10.1038/s41396-020-0668-5)
Supplement: Supplementary file 1 — Supplemental Captions [file 41396_2020_668_MOESM1_ESM.docx]

**Figure S1.** 16S rRNA tree from analyses in XCEDE on CIPRES Science Gateway

**Table S1.** Genes used to construct concatenated marker gene tree in Phylosift.

**Table S2.** Photosynthetic genes present in *A. vandensis* , Gloeobacter, and NAG Cyanobacteria. Differences between NAG Cyanobacteria and *A. vandensis* and *Gloeobacter* are indicated in green. Differences between Aurora and *Gloeobacter* are indicated in blue.

**Supplemental File 1.** Newick formatted tree for 16S rRNA analyses in XCEDE on CIPRES Science Gateway

**Supplemental File 2.** Newick formatted tree for 16S rRNA analyses in MEGA.

**Supplemental File 3.** Newick formatted tree for the IF3-C terminal.

**Supplemental File 4.** Newick formatted tree for the ribosomal L2 protein.

**Supplemental File 5.** Newick formatted tree for the D1 protein.

**Supplemental File 6.** Newick formatted tree for the concatenated marker gene tree from Phylosift.
