# Supplementary figures and images for: A phylogenetically novel cyanobacterium most closely related to Gloeobacter
Source: ISME J. 2020 May 18;14(8):2142–52. doi: 10.1038/s41396-020-0668-5 (PMC7368068; doi:10.1038/s41396-020-0668-5)

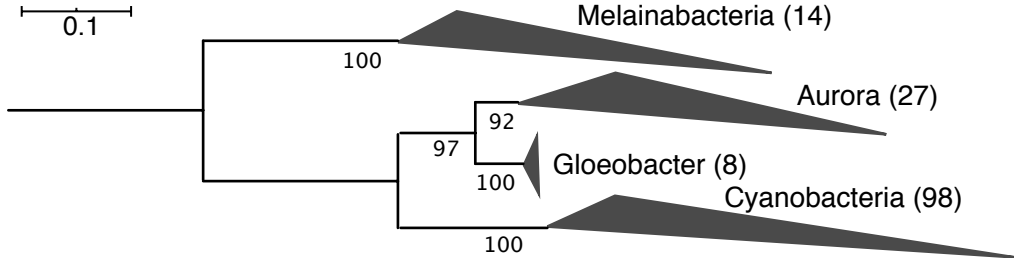

Supplement: Supplementary file 2 — Figure S1. [file 41396_2020_668_MOESM2_ESM.pdf]
