# Supplementary material for: A phylogenetically novel cyanobacterium most closely related to Gloeobacter
Source: ISME J. 2020 May 18;14(8):2142–52. doi: 10.1038/s41396-020-0668-5 (PMC7368068; doi:10.1038/s41396-020-0668-5)
Supplement: Supplementary file 5 — Supplemental File 1 [file 41396_2020_668_MOESM5_ESM.docx]

(((KC0040201_Gloeobacter_violaceus:0.00000100000050002909,KC0040181_Gloeobacter_violaceus:0.00000100000050002909):0.00000100000050002909[26],KC0040171_Gloeobacter_violaceus:0.00000100000050002909):0.00000100000050002909[76],(((NR_1217451_Gloeobacter_kilaueensis:0.00000100000050002909,EF0327841_Uncultured_Gloeobacter:0.00126975303276696946):0.01278595211902214777[100],(((((vampTnc000dn1425bprna_vamp:0.00962729425086311826,vampTncO50151209bprna_vamp:0.04780080343206559446):0.15422363123113339012[100],(vampTnc952471337bprna_vamp:0.04640210894526925617,vampTnc711091333bprna_vamp:0.01039128014384622933):0.02606165220620016224[98]):0.01725830753793939840[81],(((vampTnc586311500bprna_vamp:0.06462242651526389770,(vampTncCy2911341bprna_vamp:0.07393266548713117725,vampTncuSo261427bprna_vamp:0.05742096127577118320):0.01023114793274040174[39]):0.02370447095631436976[66],(vampTncuS1101339bprna_vamp:0.02299529020236890098,((vampTnc756931350bprna_vamp:0.05178368668233525318,vampVamChlor1446bprna_vamp:0.01173443213891950214):0.03484972306263596498[91],(vampTnc283711441bprna_vamp:0.05443440043254138549,(vampTncCy2901413bprna_vamp:0.04775438885602565492,vampTnc794621206bprna_vamp:0.19365857667955246879):0.01265941487003743524[68]):0.01552814674181929484[69]):0.02091788197364518459[49]):0.02340354256085098658[89]):0.03897507209928951361[91],vampTnc762761337bprna_vamp:0.11758657043575902745):0.03547753845072375983[83]):0.35617646735937652513[100],((cyanoTncChro61449bprna_cyano:0.16015493002666886246,((cyanoBsnTerre1415bprna_cyano:0.01608936655402617294,(cyanoBsnRober1379bprna_cyano:0.03265420538476458689,cyanoBsnAngus1479bprna_cyano:0.00548684813625290122):0.01670709329488752337[92]):0.07203460082139258314[100],(cyanoPt9Speci1434bprna_cyano:0.03907620248569158850,((((cyanoTlxSpeci1424bprna_cyano:0.01114603525323211962,cyanoCl5Speci1464bprna_cyano:0.01436637951169014195):0.05416623956613550450[100],(cyanoFoaSpeci1477bprna_cyano:0.04124487975525577971,(cyanoTncNos211693bprna_cyano:0.00000100000050002909,((cyanoTncNos161692bprna_cyano:0.00100242358673583365,((((cyanoTncNos201687bprna_cyano:0.00000100000050002909,cyanoNotSpe401438bprna_cyano:0.00123986523270856893):0.04166998448741755789[97],(((cyanoNotSpe381464bprna_cyano:0.01014827829567852870,(((cyanoNotSpe411481bprna_cyano:0.00103897588770303926,cyanoNotSpe451472bprna_cyano:0.00884466014786661724):0.01330505043257655499[99],((cyanoTncNos221687bprna_cyano:0.01893875508087169285,(cyanoNotCommu1444bprna_cyano:0.03563698024758961413,cyanoNotSpeci1481bprna_cyano:0.00879885671602996695):0.01076972999401313962[50]):0.01949875897798197116[13],cyanoTncNost21308bprna_cyano:0.03370290533597602306):0.01593487220767515408[16]):0.01717304133568893151[9],cyanoNotSpe351466bprna_cyano:0.02021742395180087248):0.01194104455439758648[8]):0.00554389737470304886[7],(cyanoNotSpe361464bprna_cyano:0.00803149851695456052,cyanoNotSpe371464bprna_cyano:0.01868939169869462263):0.00744417802634153667[76]):0.04923111702054810274[32],cyanoNotSpe431467bprna_cyano:0.00465522339672671138):0.00773070135853534072[11]):0.04027733895056317959[83],cyanoNotSphae1448bprna_cyano:0.01423780747065104060):0.02845802963301815799[32],(cyanoNotSpe421481bprna_cyano:0.01801786589171791228,(cyanoTncNos191694bprna_cyano:0.00112100741287784111,((cyanoTncNos301389bprna_cyano:0.00246916658169056948,(cyanoTncNos181688bprna_cyano:0.02943934661209318682,cyanoTncNos241389bprna_cyano:0.00181926166236268810):0.01707870299825058752[79]):0.00000100000050002909[37],cyanoTncNos251389bprna_cyano:0.00246934775489745162):0.00000100000050002909[94]):0.01055725838293881079[100]):0.00528936551731408813[56]):0.00647474138245334591[45]):0.00979890877875926568[74],(cyanoTncNos171682bprna_cyano:0.00215640308685230995,cyanoTncNos231389bprna_cyano:0.00125704977936884669):0.00000100000050002909[45]):0.00070504166092118941[40]):0.04700278242084919567[94]):0.02026012610363851080[53]):0.01444394111380971520[18],(cyanoCyuSiame1510bprna_cyano:0.03642868815255064646,(((((cyanoNdlSpeci1463bprna_cyano:0.02403150923843475756,cyanoTnide2451250bprna_cyano:0.02148309055836232495):0.02015822561913811761[62],(cyanoAbaBergi1201bprna_cyano:0.04047883108727882984,(cyanoApzOval21225bprna_cyano:0.00000100000050002909,cyanoApzOvali1225bprna_cyano:0.00000100000050002909):0.01924230025379791362[100]):0.01023137163210688372[100]):0.01517295116685301974[58],((((((((cyanoAbaSpec41368bprna_cyano:0.00000100000050002909,cyanoAbaSpec81482bprna_cyano:0.00000100000050002909):0.00000100000050002909[97],cyanoAbaSpec91429bprna_cyano:0.00000100000050002909):0.00793071857848891802[99],((cyanoAbaSpec61392bprna_cyano:0.00000100000050002909,cyanoAbaSpec71442bprna_cyano:0.00000100000050002909):0.03167558302973561696[100],cyanoAbaSpec31364bprna_cyano:0.01471629017802459326):0.00239933641692854568[43]):0.01504165704562086987[55],cyanoAbaSpec51375bprna_cyano:0.01079676815780336412):0.00241994510008100485[8],(cyanoAbaSpe231434bprna_cyano:0.00777318460297260830,(((cyanoDhpFlosa1281bprna_cyano:0.02056419284621898574,cyanoAbaAffin1256bprna_cyano:0.00688449330852280213):0.00114149593810027560[35],((cyanoAbaSmith1254bprna_cyano:0.00138231741640718487,cyanoAbaSpir71254bprna_cyano:0.00138234802639050614):0.00714079451203797778[79],(cyanoAbaSpir61307bprna_cyano:0.00000100000050002909,((cyanoAbaPlan21310bprna_cyano:0.00000100000050002909,cyanoAbaPlanc1309bprna_cyano:0.00000100000050002909):0.00412241005404921645[99],cyanoAbaSoli21311bprna_cyano:0.00440255054235453702):0.01022550186612115886[70]):0.00000100000050002909[44]):0.01254613613617857996[66]):0.02161021942966930010[96],cyanoAbaCompa1307bprna_cyano:0.00434672948723245633):0.01432362051554706035[69]):0.00250917090913309153[20]):0.04721167526804525422[100],cyanoAbaSpe101407bprna_cyano:0.01840370172234171570):0.01007572201754078529[26],(cyanoAbaSpe241435bprna_cyano:0.08230433615227691901,(cyanoSp7Torqu1411bprna_cyano:0.02201234510531553668,(cyanoAbaFlosa1307bprna_cyano:0.00000100000050002909,cyanoAbaKisse1309bprna_cyano:0.00146011708506086930):0.02163763521449096894[100]):0.03851600256808547507[100]):0.02616212245372411330[78]):0.01056345965579285132[9],cyanoNotSpe441474bprna_cyano:0.05297813449673165198):0.01053154735814813316[17]):0.01696772841922796968[33],(cyanoCaxSpec51280bprna_cyano:0.03152218463784377256,(cyanoCyuSpeci1282bprna_cyano:0.06435502759611212709,(cyanoSc6Conto1474bprna_cyano:0.07782481654175033015,((cyanoCaxSpec21474bprna_cyano:0.00884171470282131208,cyanoCaxSpec41474bprna_cyano:0.01012091820513042958):0.00671168580964191883[63],(cyanoCaxSpeci1455bprna_cyano:0.07544286862228020973,cyanoCaxSpec31474bprna_cyano:0.01882021193315166346):0.00486796979199944062[55]):0.07704988638792628630[100]):0.02795840008400441931[98]):0.02125942310228074589[42]):0.00632078324349020799[11]):0.01660317187947017020[16],(cyanoNotSpe461464bprna_cyano:0.05011095891359167165,((cyanoCp8Speci1478bprna_cyano:0.02934727604515680738,(cyanoTncBa6181462bprna_cyano:0.00000100000050002909,cyanoTncBa7581458bprna_cyano:0.00923839983084525328):0.02820883878556468619[100]):0.02000922730041619635[81],((((cyanoAbaAphan1456bprna_cyano:0.00000100000050002909,((cyanoAbaSpec21456bprna_cyano:0.00000100000050002909,cyanoAbaOsci21456bprna_cyano:0.00000100000050002909):0.00000100000050002909[18],cyanoAbaeLax21456bprna_cyano:0.00000100000050002909):0.00000100000050002909[6]):0.00000100000050002909[70],cyanoAbaIyen51456bprna_cyano:0.00000100000050002909):0.04479453975929682114[100],((((cyanoAbaFerti1456bprna_cyano:0.00000100000050002909,(cyanoAbaIyeng1456bprna_cyano:0.00000100000050002909,(cyanoAbaIyen41456bprna_cyano:0.00000100000050002909,cyanoAbaIyen21456bprna_cyano:0.00000100000050002909):0.00000100000050002909[13]):0.00000100000050002909[2]):0.00000100000050002909[0],cyanoAbaAnoma1456bprna_cyano:0.00000100000050002909):0.00000100000050002909[4],(cyanoAbaIyen31456bprna_cyano:0.00000100000050002909,cyanoAbaIyen61456bprna_cyano:0.00000100000050002909):0.00000100000050002909[6]):0.00858839394269653687[99],((cyanoAbaeLaxa1456bprna_cyano:0.00000100000050002909,cyanoAbaeLax31456bprna_cyano:0.00000100000050002909):0.00734718933803164137[100],((cyanoAbaSpir31456bprna_cyano:0.00000100000050002909,(cyanoAbaBally1456bprna_cyano:0.00000100000050002909,(cyanoAbaSpiro1456bprna_cyano:0.00000100000050002909,cyanoAbaOscil1456bprna_cyano:0.00000100000050002909):0.00000100000050002909[17]):0.00000100000050002909[9]):0.00000100000050002909[15],cyanoAbaSpir21456bprna_cyano:0.00000100000050002909):0.00982722780846206380[100]):0.00000100000050002909[44]):0.00000100000050002909[92]):0.00252324106737173912[97],cyanoNotSpe391397bprna_cyano:0.02307858506281296879):0.00733220756012008690[95]):0.01438015283441348990[52]):0.01475781109850952087[47]):0.01420337067389570648[15]):0.01325269165990737574[16]):0.02600246881457850268[69],cyanoFshSpeci1395bprna_cyano:0.13748083238381461624):0.00656353498557812106[25]):0.01523858261453290251[46]):0.02504540362057696215[87]):0.01232245323952670980[48],(cyanoTncOsc101311bprna_cyano:0.05244968081544210126,(cyanoTncChro41313bprna_cyano:0.03122686695829473766,((cyanoTncBa3691311bprna_cyano:0.07802010508930735944,cyanoTncBa4201310bprna_cyano:0.05761545907827672619):0.00900734506237914172[35],cyanoTncChro51311bprna_cyano:0.07856085118649971943):0.01217657765778151942[30]):0.00877228967867224596[38]):0.02169250585378780169[44]):0.13440648637177016189[100]):0.06821989442733950026[97],((KF8564871_Uncultured_cyanobacterium_clone_23BF24C_16S_ribosomal_RNA_gene_partial_sequence:0.00383530163024147115,(JQ7930081_Uncultured_cyanobacterium_clone_Blake1cm57_16S_ribosomal_RNA_partial_sequence:0.00344522666697447914,JQ7930001_Uncultured_cyanobacterium_clone_Blake1cm5_16S_ribosomal_RNA_partial_sequence:0.00000100000050002909):0.00617852795530013637[100]):0.10178337516364104054[100],(KR9232911_Uncultured_cyanobacterium_clone_OTU_425_16S_ribosomal_RNA_gene_partial_sequence:0.02550833218876865163,(KR9232981_Uncultured_cyanobacterium_clone_OTU_518_16S_ribosomal_RNA_gene_partial_sequence:0.00000100000050002909,((KU2225291_Uncultured_bacterium_clone_OTU_2403_16S_ribosomal_RNA_gene_partial_sequence:0.00000100000050002909,(((KM1537501_Uncultured_bacterium_clone_LNH_1_16_12_Pumice229449_16S_ribosomal_RNA_gene_partial_sequence:0.01139336286940395571,(KM1500011_Uncultured_bacterium_clone_LNH_1_16_12_Pumice107561_16S_ribosomal_RNA_gene_partial_sequence:0.00000100000050002909,KM1523471_Uncultured_bacterium_clone_LNH_1_16_12_Pumice180710_16S_ribosomal_RNA_gene_partial_sequence:0.00000100000050002909):0.00000100000050002909[65]):0.01292697916461069130[68],(KM1497401_Uncultured_bacterium_clone_LNH_1_16_12_Pumice99444_16S_ribosomal_RNA_gene_partial_sequence:0.03584759242371519244,(KM1467651_Uncultured_bacterium_clone_LNH_1_16_12_Pumice3278_16S_ribosomal_RNA_gene_partial_sequence:0.04262934362814231604,(KM1473021_Uncultured_bacterium_clone_LNH_1_16_12_Pumice21055_16S_ribosomal_RNA_gene_partial_sequence:0.00925386549715134614,(KM1495761_Uncultured_bacterium_clone_LNH_1_16_12_Pumice94702_16S_ribosomal_RNA_gene_partial_sequence:0.00985255922528694740,KM1524881_Uncultured_bacterium_clone_LNH_1_16_12_Pumice185343_16S_ribosomal_RNA_gene_partial_sequence:0.00509518107795328690):0.00562420290248693416[31]):0.01345087215795541524[32]):0.04220135784867044826[53]):0.00780093077245445736[50]):0.01820370612466032378[81],KR9232741_Uncultured_cyanobacterium_clone_OTU_263_16S_ribosomal_RNA_gene_partial_sequence:0.03679418432745946987):0.06464974152969792109[97]):0.03783762414303849103[81],(((JQ3070821_Uncultured_bacterium_clone_9_1_16S_ribosomal_RNA_gene_partial_sequence:0.00182835331980859693,(JQ3070911_Uncultured_bacterium_clone_9_11_16S_ribosomal_RNA_gene_partial_sequence:0.00183042573608289548,JQ3070841_Uncultured_bacterium_clone_9_3_16S_ribosomal_RNA_gene_partial_sequence:0.00920496881395664428):0.00000100000050002909[39]):0.00000100000050002909[31],JQ3070851_Uncultured_bacterium_clone_9_4_16S_ribosomal_RNA_gene_partial_sequence:0.00000100000050002909):0.00000100000050002909[56],(((((JQ3070901_Uncultured_bacterium_clone_9_10_16S_ribosomal_RNA_gene_partial_sequence:0.00734590457514420168,(JQ3070921_Uncultured_bacterium_clone_9_12_16S_ribosomal_RNA_gene_partial_sequence:0.00182633568234059126,JQ3070881_Uncultured_bacterium_clone_9_8_16S_ribosomal_RNA_gene_partial_sequence:0.00182658786483822790):0.00000100000050002909[22]):0.00000100000050002909[2],(JQ3070871_Uncultured_bacterium_clone_9_7_16S_ribosomal_RNA_gene_partial_sequence:0.00182686034618206807,JQ3070891_Uncultured_bacterium_clone_9_9_16S_ribosomal_RNA_gene_partial_sequence:0.00182654388508959788):0.00000100000050002909[19]):0.00182549237540500522[62],AB6306821_Uncultured_bacterium_gene_for_16S_ribosomal_RNA_partial_sequence_clone_MPB26:0.00000100000050002909):0.00000100000050002909[28],AB6306821_U:0.00000100000050002909):0.00000100000050002909[59],AuroraVandensis_k99_473239:0.00000100000050002909):0.00000100000050002909[60]):0.00941969332258571317[94]):0.02273878079344371708[76]):0.03647677288627204639[91]):0.06343710990867101884[100]):0.03929571024364832132[92]):0.04409876028310257684[100]):0.01100460933178850145[98],(NR_0742821_Gloeobacter_violaceus:0.00000100000050002909,KM0200091_Gloeobacter_violaceus:0.00000100000050002909):0.00124030636446985517[72]):0.00122092359258495469[69],FR7989241_Gloeobacter_violaceus:0.00124659181771889218);
