# Supplementary material for: A phylogenetically novel cyanobacterium most closely related to Gloeobacter
Source: ISME J. 2020 May 18;14(8):2142–52. doi: 10.1038/s41396-020-0668-5 (PMC7368068; doi:10.1038/s41396-020-0668-5)
Supplement: Supplementary file 6 — Supplemental File 2. [file 41396_2020_668_MOESM6_ESM.docx]

((((((((((((((((((((((cyanoNotSphae1448bprna_cyano:0.00000000,cyanoNotSpe431467bprna_cyano:0.00000000)0.2300:0.00000000,cyanoNotSpe351466bprna_cyano:0.00000000)0.4100:0.00000000,cyanoNotSpeci1481bprna_cyano:0.00620302)0.2800:0.00309293,cyanoTncNos201687bprna_cyano:0.00000000)0.3400:0.00309389,cyanoNotSpe401438bprna_cyano:0.00000000)0.1700:0.00309415,(cyanoTncNos301389bprna_cyano:0.00000000,(cyanoTncNos181688bprna_cyano:0.00000000,(cyanoTncNos191694bprna_cyano:0.00000000,(cyanoTncNos221687bprna_cyano:0.00000000,(cyanoTncNos241389bprna_cyano:0.00000000,cyanoTncNos251389bprna_cyano:0.00000000)0.0600:0.00000000)0.0400:0.00000000)0.0000:0.00000000)0.0400:0.00000000)0.2900:0.00000000)0.3300:0.00309505,(cyanoNotSpe451472bprna_cyano:0.00620795,(cyanoNotSpe381464bprna_cyano:0.00310295,cyanoNotSpe411481bprna_cyano:0.00000000)0.4500:0.00000000)0.4700:0.00000000)0.1100:0.00309997,cyanoNotCommu1444bprna_cyano:0.00311156)0.3800:0.00307901,cyanoNotSpe461464bprna_cyano:0.00624292)0.2900:0.00622471,(cyanoAbaSpec61392bprna_cyano:0.00000000,((((cyanoAbaBergi1201bprna_cyano:0.00311571,cyanoApzOvali1225bprna_cyano:0.00620552)0.6500:0.00301550,cyanoNdlSpeci1463bprna_cyano:0.00941089)0.8100:0.01568206,cyanoAbaSpec71442bprna_cyano:0.00000000)0.0100:0.00000000,(cyanoAbaSpe231434bprna_cyano:0.02210730,(cyanoAbaSpec41368bprna_cyano:0.00000000,(cyanoAbaSpec81482bprna_cyano:0.00000000,cyanoAbaSpec91429bprna_cyano:0.00000000)0.3200:0.00000000)0.9300:0.00000000)0.7800:0.01875628)0.0000:0.00000000)0.1200:0.00623104)0.0400:0.00575173,cyanoTncBa7581458bprna_cyano:0.02123046)0.0000:0.00731765,(cyanoFoaSpeci1477bprna_cyano:0.01249524,(cyanoAbaSpe101407bprna_cyano:0.00937402,(cyanoTncNos231389bprna_cyano:0.00309399,((cyanoNotSpe371464bprna_cyano:0.00309372,cyanoNotSpe421481bprna_cyano:0.00309795)0.0200:0.00000000,(cyanoNotSpe361464bprna_cyano:0.00000000,(cyanoTncNos161692bprna_cyano:0.00000000,(cyanoTncNos171682bprna_cyano:0.00000000,cyanoTncNos211693bprna_cyano:0.00000000)0.1100:0.00000000)0.0700:0.00000000)0.2900:0.00000000)0.1100:0.00000000)0.7400:0.00309982)0.4000:0.00310489)0.1500:0.00327527)0.0100:0.00311390,(cyanoNotSpe441474bprna_cyano:0.01250027,(cyanoCyuSiame1510bprna_cyano:0.00621428,(((((cyanoAbaeLax21456bprna_cyano:0.00000000,cyanoAbaIyen51456bprna_cyano:0.00000000)0.1800:0.00000000,cyanoAbaAphan1456bprna_cyano:0.00000000)0.0700:0.00000000,cyanoAbaSpec21456bprna_cyano:0.00000000)0.1000:0.00000000,cyanoAbaOsci21456bprna_cyano:0.00000000)1.0000:0.01562450,((cyanoAbaeLaxa1456bprna_cyano:0.00000000,cyanoAbaeLax31456bprna_cyano:0.00000000)0.6200:0.00310055,((((((cyanoAbaSpir21456bprna_cyano:0.00000000,cyanoAbaSpir31456bprna_cyano:0.00000000)0.1400:0.00000000,cyanoAbaOscil1456bprna_cyano:0.00000000)0.0600:0.00000000,cyanoAbaBally1456bprna_cyano:0.00000000)0.1200:0.00000000,cyanoAbaSpiro1456bprna_cyano:0.00000000)0.5600:0.00309266,cyanoNotSpe391397bprna_cyano:0.00000000)0.4200:0.00000000,(cyanoAbaIyeng1456bprna_cyano:0.00000000,(cyanoAbaAnoma1456bprna_cyano:0.00000000,(cyanoAbaIyen21456bprna_cyano:0.00000000,(cyanoAbaIyen31456bprna_cyano:0.00000000,(cyanoAbaIyen41456bprna_cyano:0.00000000,(cyanoAbaIyen61456bprna_cyano:0.00000000,cyanoAbaFerti1456bprna_cyano:0.00000000)0.1100:0.00000000)0.0300:0.00000000)0.0300:0.00000000)0.0200:0.00000000)0.0500:0.00000000)0.6500:0.00310055)0.5600:0.00000000)0.8000:0.00000000)0.9600:0.00622076)0.2600:0.00307880)0.2500:0.00600585)0.0000:0.00000000,(cyanoTlxSpeci1424bprna_cyano:0.00000000,(cyanoCl5Speci1464bprna_cyano:0.00047384,cyanoCp8Speci1478bprna_cyano:0.01880118)0.3100:0.00619329)0.1000:0.00328996)0.0200:0.00460158,((cyanoSp7Torqu1411bprna_cyano:0.00712398,cyanoAbaSpe241435bprna_cyano:0.04760587)0.7600:0.03094611,(cyanoSc6Conto1474bprna_cyano:0.02535336,(cyanoCaxSpec31474bprna_cyano:0.00318995,(cyanoCaxSpeci1455bprna_cyano:0.03501128,(cyanoCaxSpec21474bprna_cyano:0.00639721,cyanoCaxSpec41474bprna_cyano:0.01554525)0.3700:0.00630732)0.2300:0.00316152)0.9900:0.03878421)0.8100:0.01580282)0.3000:0.01866011)0.0200:0.00637084,cyanoFshSpeci1395bprna_cyano:0.03964852)0.1700:0.00787025,cyanoPt9Speci1434bprna_cyano:0.00627611)0.4200:0.01039305,(cyanoBsnTerre1415bprna_cyano:0.00568861,(cyanoBsnRober1379bprna_cyano:0.00619774,cyanoBsnAngus1479bprna_cyano:0.00000000)0.7200:0.00361991)0.8500:0.01540450)0.7300:0.03167044,cyanoTncChro61449bprna_cyano:0.03402481)0.9900:0.10289508,(NR_121745.1_Gloeobacter_kilaueensis_strain_JS1_16S_ribosomal_RNA_complete_sequence:0.00000000,(NR_074282.1_Gloeobacter_violaceus_strain_PCC_7421_16S_ribosomal_RNA_complete_sequence:0.00000000,(FR798924.1_Gloeobacter_violaceus_VP3-01_partial_16S_rRNA_gene_strain_VP3-01:0.00000000,(KM020009.1_Gloeobacter_violaceus_SAG_35.87_16S_ribosomal_RNA_gene_partial_sequence:0.00000000,(KC004017.1_Gloeobacter_violaceus_CCALA_981_16S_ribosomal_RNA_gene_partial_sequence:0.00000000,(KC004018.1_Gloeobacter_violaceus_CCALA_980_16S_ribosomal_RNA_gene_partial_sequence:0.00000000,KC004020.1_Gloeobacter_violaceus_PCC_9601_16S_ribosomal_RNA_gene_partial_sequence:0.00000000)0.1100:0.00000000)0.0200:0.00000000)0.0600:0.00000000)0.0700:0.00000000)0.8400:0.00620660)0.9900:0.02450837)0.2100:0.01948951,(((JQ793008.1_Uncultured_cyanobacterium_clone_Blake1cm57_16S_ribosomal_RNA_partial_sequence:0.00000000,KF856487.1_Uncultured_cyanobacterium_clone_23BF24C_16S_ribosomal_RNA_gene_partial_sequence:0.00000000)0.4500:0.00000000,JQ793000.1_Uncultured_cyanobacterium_clone_Blake1cm5_16S_ribosomal_RNA_partial_sequence:0.00000000)0.9900:0.04505899,((KM146765.1_Uncultured_bacterium_clone_LNH_1_16_12_Pumice.3278_16S_ribosomal_RNA_gene_partial_sequence:0.00783105,KR923274.1_Uncultured_cyanobacterium_clone_OTU_263_16S_ribosomal_RNA_gene_partial_sequence:0.02024428)0.8800:0.01655294,(KR923298.1_Uncultured_cyanobacterium_clone_OTU_518_16S_ribosomal_RNA_gene_partial_sequence:0.01195303,(AuroraVandensis_green_k99_473239:0.00000000,JQ307091.1_Uncultured_bacterium_clone_9_11_16S_ribosomal_RNA_gene_partial_sequence:0.00307604)0.9800:0.00987761)0.8700:0.02105107)1.0000:0.04830068)0.6800:0.01601713)0.7300:0.06757373,(vampTnc283711441bprna_vamp:0.01740103,(vampVamChlor1446bprna_vamp:0.02568174,(vampTncuSo261427bprna_vamp:0.01565133,vampTnc586311500bprna_vamp:0.01259437)0.9600:0.01305693)0.8900:0.02743377)1.0000:0.10074247,NR_114308.1_Brevundimonas_abyssalis_strain_TAR-001_16S_ribosomal_RNA_gene_partial_sequence:0.18908247);
