# Supplementary material for: A phylogenetically novel cyanobacterium most closely related to Gloeobacter
Source: ISME J. 2020 May 18;14(8):2142–52. doi: 10.1038/s41396-020-0668-5 (PMC7368068; doi:10.1038/s41396-020-0668-5)
Supplement: Supplementary file 7 — Supplemental File 3. [file 41396_2020_668_MOESM7_ESM.docx]

(brevundimo:0.509701082637139,(((AuroraVandensisGreen|sourc:0.00000100000050002909,AuroraVandensisPurple|sourc:0.00000100000050002909):0.41137550077618539879[100],((kilaueensi:0.04522191742011644894,violaceus|:0.03332370623739220467):0.28616983390566208545[100],((((fischerell:0.00877021979905035984,((scytonema|:0.02659941855533061450,(nostocales:0.01181278420935892928,mastigocla:0.02622914725036871314):0.00000100000050002909[37]):0.02727009649049560655[76],((nostocpunc:0.03698078481205734069,(tolypothri:0.00000100000050002909,aulosirala:0.00000100000050002909):0.01459678554591359312[100]):0.00835574803141799632[54],bin_id_cal:0.03825259262091345452):0.00852350037526942300[47]):0.03425000317143349349[76]):0.08960329748600752431[95],((moorea|sou:0.08311977226262894092,((prochloroc:0.41656306324823144660,spirulina|:0.16225385588274238158):0.02865288472423667063[19],(cyanothece:0.11829582992876566672,(microcisti:0.06307932044366983126,bin_id_ple:0.10277009417029955851):0.01831426581114286439[20]):0.05496833472954589656[46]):0.01700033426759617675[8]):0.06234883544844321140[17],((acaryochlo:0.25175887445706712775,pseudanaba:0.20495226775701030908):0.06737273612835849301[40],geitlerine:0.11496954114185818641):0.04671672593576260690[21]):0.01312060034267900112[2]):0.02452456718001763680[5],((planktothr:0.13251087350968310252,cyanobacte:0.30350202738842130268):0.07234420212957315410[39],bin_id_lep:0.15783298120209909854):0.00980793575248929336[3]):0.04473238503200932525[23],chamaesiph:0.18981445666109747483):0.16870243309477833682[76]):0.10982306926624704757[67]):0.27911199165779276843[99],((bin_id_sbm:0.48900923608826901701,(glnubmers|:0.28641195342911651700,hgwmelaina:0.19036359265907978888):0.46757546076652084510[100]):0.25069817385759968209[94],((((mh|source_:0.01465946109262752407,melainabac:0.00000100000050002909):0.11568631924701892888[100],(((humthree|s:0.00000100000050002909,(cfd279907c:0.00000100000050002909,(((((07dfd5d4a4:0.00000100000050002909,humthirtee:0.00000100000050002909):0.00000100000050002909[1],(humeightee:0.00000100000050002909,humeight|s:0.00000100000050002909):0.00000100000050002909[17]):0.00000100000050002909[0],((humten|sou:0.00000100000050002909,humfifteen:0.00000100000050002909):0.00000100000050002909[2],humninetee:0.00000100000050002909):0.00000100000050002909[5]):0.00000100000050002909[0],humtwentyt:0.00000100000050002909):0.00000100000050002909[0],humsixteen:0.00000100000050002909):0.00000100000050002909[0]):0.00000100000050002909[0]):0.00000100000050002909[18],humsevente:0.00000100000050002909):0.13490928828222986668[100],((humseven|s:0.00000100000050002909,humtwelve|:0.00000100000050002909):0.08997279976053666750[100],zag|source:0.06195308832569276086):0.07684698834896412645[83]):0.10940340152222706449[92]):0.28911816073640300706[99],(gwa|source:0.24498112964951898607,bin_id_gwf:0.15053542684939333340):0.20984026709379277609[98]):0.32700389376806776598[98],(vamp|sourc:1.02357948175635105059,(rifeltwelv:0.00000100000050002909,rifeltwo|s:0.00000100000050002909)OROOT:0.56226303145703993014[100]):0.16334821853082803766[67]):0.12103381350967561125[33]):0.10237478474210012780[50]):0.509701082637139);
