# Supplementary material for: A phylogenetically novel cyanobacterium most closely related to Gloeobacter
Source: ISME J. 2020 May 18;14(8):2142–52. doi: 10.1038/s41396-020-0668-5 (PMC7368068; doi:10.1038/s41396-020-0668-5)
Supplement: Supplementary file 8 — Supplemental File 4. [file 41396_2020_668_MOESM8_ESM.docx]

(brevundimo:0.560319287634088,((glnubmers|:0.17229736814904589304,hgwmelaina:0.16149468867942295969):0.09842775624121312406[88],(sbmnraac|s:0.28576307032982234402,((((AuroraVandensisPurple|sourc:0.00000100000050002909,AuroraVandensisGreen|sourc:0.00000100000050002909):0.26262841942562875630[100],(kilaueensi:0.05022932065563878723,violaceus|:0.00929964889247129022):0.15403231201439329667[100]):0.11821755110104488484[86],((bin_id_spi:0.19270717581010346486,((bin_id_cya:0.10148990203008238786,(pleurocaps:0.06932897796540445079,microcisti:0.12211248215890195545):0.06305999182921281920[92]):0.05803684675327323156[53],cyanobacte:0.25011764251841506201):0.03307780844630901079[61]):0.06617799613551784976[50],((leptolynby:0.17015116887329645445,(moorea|sou:0.09264249157720004513,(planktothr:0.16942943709118388207,(((chamaesiph:0.17999586284901289424,(calothrixs:0.05816072371801984942,(nostocpunc:0.06024696465467916512,((tolypothri:0.00000100000050002909,aulosirala:0.00000100000050002909):0.04400820827032352750[100],(fischerell:0.05184659280717100749,((bin_id_nos:0.00000100000050002909,mastigocla:0.05534601923087362207):0.02843137110470395773[69],scytonema|:0.04183600103676928667):0.03565611697509766392[79]):0.06315988181337213458[93]):0.03515602764482565756[53]):0.03107352478846390348[71]):0.02099172471390423414[42]):0.02513502374055336802[54],(acaryochlo:0.24174188822790917963,pseudanaba:0.26212782139344859011):0.06568875373223898029[81]):0.05013843685948941170[81],geitlerine:0.16937510021906709845):0.02329297676613969678[40]):0.01917766375608596224[30]):0.02784317909997095775[37]):0.04820522760612236390[43],prochloroc:0.43804295952686683746):0.02567069315471653262[30]):0.11541763681568091571[77]):0.18249291572840609099[95],(((rifeltwo|s:0.00000100000050002909,rifeltwelv:0.00000100000050002909):0.57933488819399381331[100],vamp|sourc:0.38382273627369889457):0.08980485833658193029[50],(gwff|sourc:0.13659946682281223773,((((humeightee:0.00000100000050002909,((humtwentyt:0.00000100000050002909,((bin_id_hum:0.00000100000050002909,(((((((humsevente:0.00000100000050002909,humeight|s:0.02871860706637732394):0.00000100000050002909[6],humeleven|:0.00000100000050002909):0.00000100000050002909[0],humten|sou:0.00000100000050002909):0.00000100000050002909[3],humfifteen:0.00000100000050002909):0.00000100000050002909[0],humfour|so:0.00000100000050002909):0.00000100000050002909[0],humninetee:0.00000100000050002909):0.00000100000050002909[0],humsixteen:0.00000100000050002909):0.00000100000050002909[0]):0.00000100000050002909[0],humthirtee:0.00000100000050002909):0.00000100000050002909[0]):0.00000100000050002909[4],humfive|so:0.00000100000050002909):0.00000100000050002909[14]):0.04117759976002557942[100],((zagone|sou:0.00000100000050002909,(mh|source_:0.00000100000050002909,melainabac:0.00000100000050002909):0.00849792148281613285[84]):0.01906979805066376377[88],((humseven|s:0.00000100000050002909,humtwelve|:0.00000100000050002909):0.01487748615015125481[100],bin_id_zag:0.04557774481223917512):0.02557601795484422055[78]):0.01930189407315812886[86]):0.23841170177501447291[100],(riflethirt:0.00000100000050002909,gwf|source:0.00000100000050002909):0.23534160588041735940[100]):0.03476951437557783409[36],gwa|source:0.20099649343743553054)OROOT:0.07351904988865430590[55]):0.08190569474944366102[59]):0.12744022202514021513[77]):0.11981348305861473647[91]):0.22837434857303226532[95]):0.560319287634088);
