# Supplementary material for: A phylogenetically novel cyanobacterium most closely related to Gloeobacter
Source: ISME J. 2020 May 18;14(8):2142–52. doi: 10.1038/s41396-020-0668-5 (PMC7368068; doi:10.1038/s41396-020-0668-5)
Supplement: Supplementary file 9 — Supplemental File 5. [file 41396_2020_668_MOESM9_ESM.docx]

(((((507_Prochlorothrix_hollandica:0.15760358807488369925,((((429_Cyanothece_sp._PCC_7425:0.10186133053888209166,417_Synechococcus_sp._PCC_6312:0.01075551440872886053):0.03848058708629984587[18],(413_Thermosynechococcus_sp._NK:0.01076315849794050490,418_Thermosynechococcus_elonga:0.00527546703529301834):0.01113951182838670076[82]):0.02788725479750946146[18],(397_Synechococcus_sp._JA-3-3Ab:0.02140082765467733392,396_Synechococcus_sp._JA-2-3B:0.01846307126868128312):0.08505280848402060889[92]):0.02003621769073095013[5],((((((444_Synechococcus_sp._RCC307:0.03872439253578095764,((474_Synechococcus_sp._WH_8109:0.00000100000050002909,(477_Synechococcus_sp._BL107:0.00000100000050002909,479_Synechococcus_sp._CC9902:0.00000100000050002909):0.02746148321537328352[97]):0.00905861259585800938[30],((475_Synechococcus_sp._WH_8102:0.00539553251013804788,((((500_Synechococcus_sp._WH_8016:0.00000100000050002909,487_Synechococcus_sp._WH_8016:0.00000100000050002909):0.00000100000050002909[76],494_Synechococcus_sp._CC9311:0.00538082746730995758):0.02434977403312587121[98],((((550_Prochlorococcus_sp._W2:0.01415255299400869013,(548_Prochlorococcus_marinus_su:0.01483337032032082045,(545_Prochlorococcus_marinus_st:0.00000100000050002909,546_Prochlorococcus_marinus_st:0.00492272791305344969):0.00000100000050002909[66]):0.01059252446465368952[66]):0.05981215695880609529[98],((551_Prochlorococcus_marinus_st:0.00527732244527434786,552_Prochlorococcus_marinus_st:0.00443245170055174553):0.06607423675687536246[98],((538_Prochlorococcus_marinus:0.00507331235719412840,540_Prochlorococcus_marinus_su:0.00000100000050002909):0.00496531967631669811[98],537_Prochlorococcus_marinus_st:0.04058502193691885390):0.03882558490316771749[76]):0.02446806897512010179[55]):0.15690269151642532997[78],(525_Prochlorococcus_marinus_st:0.00000100000050002909,534_Prochlorococcus_marinus_st:0.00548342331928977720):0.09593888617038534572[100]):0.10146384547796664932[76],472_Synechococcus_sp._RS9916:0.00539146531982687225):0.00000100000050002909[16]):0.00000100000050002909[7],463_Synechococcus_sp._RS9917:0.00000100000050002909):0.01433096671048613747[18]):0.00000100000050002909[6],467_Synechococcus_sp._WH_7803:0.00000100000050002909):0.00000100000050002909[6]):0.03729501817169128541[35]):0.00765190028915206134[3],((483_Cyanobium_gracile_PCC_6307:0.02700283955264837568,473_Synechococcus_sp._WH_5701:0.00000100000050002909):0.01792963103008732295[78],455_Cyanobium_sp._PCC_7001:0.00914254364448358406):0.01442821242508261770[49]):0.06253364601398524181[59],(443_Synechococcus_sp.:0.00545810566332571856,(433_Synechococcus_elongatus_PC:0.00000100000050002909,441_Synechococcus_elongatus_PC:0.00546131990818232629):0.00000100000050002909[33]):0.06204823175003623681[100]):0.01841217534923909882[34],((((469_Synechococcus_sp._PCC_7002:0.00000100000050002909,496_Leptolyngbya_sp._PCC_7376:0.05550868304728809322):0.05572143482973571788[98],(442_filamentous_cyanobacterium:0.03587153855694448501,523_Geitlerinema_sp._PCC_7105:0.11125203245608276947):0.02380290140194689374[58]):0.04021816846089454334[30],(470_Spirulina_subsalsa:0.05047975375453932212,((480_Rubidibacter_lacunae_KORDI:0.01085469691018150877,486_Rubidibacter_lacunae_KORDI:0.00000100000050002909):0.03526055050289925857[97],(419_Halothece_sp._PCC_7418:0.00950359209034650626,(440_Dactylococcopsis_salina_PC:0.00000100000050002909,435_Dactylococcopsis_salina_PC:0.00534116029403764250):0.02837938914861151685[100]):0.07770066732825368916[100]):0.03540330878903906464[82]):0.02716914098040799919[50]):0.01875310138897550230[20],(((426_Pleurocapsa_sp._PCC_7327:0.03850469706963650929,((421_Microcoleus_sp._PCC_7113:0.06753483697095756522,(432_Stanieria_cyanosphaera_PCC:0.01292774266130712092,(509_Pleurocapsa_sp._PCC_7319:0.00000100000050002909,515_Pleurocapsa_sp._PCC_7319:0.00535414772685612050):0.11493285362027653995[100]):0.02885404634343862601[81]):0.02660321691393705348[36],(476_Cyanothece_sp._PCC_7822:0.03788187981528558929,405_Cyanothece_sp._PCC_7424:0.01910512733070625357):0.01592843055758590143[87]):0.01628680915572568513[19]):0.03483186646958021104[58],(((449_Cyanobacterium_aponinum_PC:0.00653073626507187990,422_Cyanobacterium_aponinum_PC:0.01061269625871792514):0.02968453312287940710[96],(478_Geminocystis_herdmanii:0.04443414161370896898,439_Cyanobacterium_stanieri_PC:0.03467379799077693892):0.02513348204024907009[46]):0.02014754528380511764[51],(438_Moorea_producens_3L:0.12008656134924504399,((((414_Cyanothece_sp._CCY0110:0.00556653696921244161,415_Cyanothece_sp._ATCC_51142:0.00000100000050002909):0.00000100000050002909[20],406_Cyanothece_sp._PCC_8802:0.02201042688791583421):0.00000100000050002909[2],420_Cyanothece_sp._ATCC_51142:0.00000100000050002909):0.02394638843820133881[87],412_Crocosphaera_watsonii_WH_8:0.00552604408259571491):0.01122951821849541877[76]):0.02169478695101361571[8]):0.01977891949000220792[1]):0.01818474285679967661[1],(457_Synechocystis_sp._PCC_6803:0.03923075562197161220,468_Microcystis_aeruginosa_SPC:0.04981020977284892487):0.04156383050705916155[50]):0.00564401552452513215[0]):0.04211056237790358064[6]):0.02708572441907202560[0],((((533_Acaryochloris_marina_MBIC1:0.00527485086598899772,531_Acaryochloris_sp._CCMEE_54:0.01107339174844655834):0.06104118243318736731[100],541_Acaryochloris_marina_MBIC1:0.05972335115691705665):0.12339665008923839107[98],(((((399_Gloeobacter_violaceus_PCC_:0.04601938628105173290,403_Gloeobacter_kilaueensis_JS:0.03909028452033996848):0.29534697523625569326[99],453_Synechococcus_sp._PCC_7336:0.11397624343443216177):0.01139289847234641051[3],(514_Nodosilinea_nodulosa:0.08106390848441857866,(504_Leptolyngbya_sp._PCC_7375:0.00722671942715363835,503_Leptolyngbya_sp._Heron_Isl:0.00378147953540893152):0.05290153187578719440[97]):0.02027959507921090962[38]):0.02910737882212539873[5],454_Synechococcus_sp._PCC_7335:0.07876318835055276524):0.00801653563866423606[0],((544_Pseudanabaena_sp._PCC_7367:0.14332260861561041398,532_Pseudanabaena_sp._PCC_6802:0.08206006917173989945):0.03546699049056601522[45],(535_Synechococcus_sp._PCC_7502:0.05247886945664409497,543_Pseudanabaena_biceps_PCC_7:0.09068156613966678326):0.04073886620907101602[81]):0.07528300017451500237[56]):0.01854535210154524202[0]):0.00678252574809530984[0],(((495_Leptolyngbya_boryana:0.08548785160733199384,428_Oscillatoriales_cyanobacte:0.03457256701788956332):0.04966932964362971714[86],((((510_Chamaesiphon_minutus_PCC_6:0.17823847442578250044,502_Crinalium_epipsammum_PCC_9:0.05195019900226525295):0.04949435956072836124[31],(446_Oscillatoria_acuminata_PCC:0.00550837452889162311,448_Oscillatoria_acuminata_PCC:0.00000100000050002909):0.07230717458621713223[100]):0.02646242241970106837[6],464_Oscillatoria_sp._PCC_10802:0.04553124825264514425):0.01192974751199340003[3],((459_Chroococcidiopsis_thermali:0.10326102579360943445,(524_Oscillatoria_formosa:0.03871761016866577532,((522_Microcoleus_vaginatus_FGP-:0.00000100000050002909,520_Microcoleus_vaginatus_FGP-:0.00534304013310176774):0.01069691959497743282[88],529_Oscillatoria_nigro-viridis:0.00000100000050002909):0.04204753444636968179[94]):0.01111425772677948447[55]):0.02263513189602230427[43],(528_Trichodesmium_erythraeum_I:0.11255585278224843060,(489_Lyngbya_sp._PCC_8106:0.07098351822753776186,(484_Arthrospira_platensis_C1:0.00543321530675254689,482_Arthrospira_platensis_str.:0.00000100000050002909):0.02062889664391161226[100]):0.04219172158346072565[88]):0.04609209714402561636[85]):0.02897545915226746455[31]):0.01851163167863527706[4]):0.03503273465534963160[6],(436_Coleofasciculus_chthonopla:0.09985064520929104459,425_Geitlerinema_sp._PCC_7407:0.06625749714707170057):0.03296050041932541835[28]):0.01399309424694469318[1]):0.01209787740496578133[0]):0.02234194635606152890[0],(((434_Fischerella_sp._JSC-11:0.00000100000050002909,(501_Fischerella_muscicola:0.00000100000050002909,447_Fischerella_sp._PCC_9339:0.01121813030549989945):0.00555433255031874493[37]):0.00000100000050002909[48],(430_Richelia_intracellularis_H:0.00000100000050002909,437_Richelia_intracellularis_H:0.00535301766387726290):0.06008931713565437982[97]):0.01593278689724376626[29],((431_cyanobacterium_PCC_7702:0.00000100000050002909,398_cyanobacterium_PCC_7702:0.00000100000050002909):0.02863412240232265926[100],((409_Mastigocladopsis_repens:0.01095343943688030681,(((408_Rivularia_sp._PCC_7116:0.06201635640804812472,465_Calothrix_sp._PCC_6303:0.02870912350115202571):0.01651655650023054964[11],(452_Nostoc_sp._PCC_7524:0.00000100000050002909,(458_Anabaena_variabilis_ATCC_2:0.00000100000050002909,(481_Nostoc_sp._PCC_7120:0.00000100000050002909,(((466_Calothrix_sp._PCC_7507:0.02865420986781448687,456_Microchaete_sp._PCC_7126:0.01645419747174296093):0.05099588643685677714[98],485_Nodularia_spumigena_CCY941:0.05318759140207354741):0.03211556888838033169[38],(488_Nostoc_punctiforme_PCC_731:0.00558394738140989295,((((493_Anabaena_sp._90:0.03369024352985763859,(499_Anabaena_sp._PCC_7108:0.00000100000050002909,(508_Nostoc_azollae_0708:0.01688520705915843786,512_Anabaena_cylindrica_PCC_71:0.02249964408704858629):0.01117551233107958245[22]):0.01703831969922754708[39]):0.01104187652495029412[14],471_Raphidiopsis_brookii_D9:0.02262117620734087689):0.00000100000050002909[11],461_Cylindrospermum_stagnale_P:0.01639947403024420713):0.01658393757191655229[23],423_Nostoc_sp._PCC_7107:0.02189478821030384562):0.01099771505188884037[4]):0.00000100000050002909[0]):0.01088244402582534513[5]):0.01633404557113136632[6]):0.01639875719640060056[12]):0.02127013083922726921[13]):0.00604817595078124132[1],407_Calothrix_sp._PCC_7103:0.01658658214127939345):0.00000100000050002909[0]):0.01254936844681890304[1],460_Chlorogloeopsis_fritschii:0.01771050858480499243):0.01704432416372788500[2]):0.02561520605149849791[1]):0.04104797003392623295[5]):0.01638606143248331690[1]):0.01989471421987961189[4]):0.07772904857089751873[41],539_Oscillatoriales_cyanobacte:0.17824100476375015489):0.02575384425598602200[23],(542_Synechococcus_sp._PCC_7335:0.30207166030999410555,516_Pleurocapsa_sp._PCC_7327:0.19630476987915651543):0.07440235104863014703[66]):0.06987285390965883303[30],(519_Calothrix_sp._PCC_7507:0.13692035735571103650,(517_Chroococcidiopsis_thermali:0.12785617927359105295,(530_Chlorogloeopsis_fritschii:0.03926235142283977103,(490_Fischerella_sp._JSC-11:0.02816991547915689148,518_Fischerella_muscicola:0.00575831934879981328):0.03540934442414038485[67]):0.04241251734091582015[69]):0.06466412740627351663[73]):0.00000100000050002909[56]):2.48205562223016[100],((497_Gloeobacter_kilaueensis_JS:1.54325514956781706388,(363_Pleurocapsa_sp._PCC_7327:0.39488760119098592627,(385_Synechococcus_sp._PCC_7335:0.97680107130810855942,(339_Chroococcidiopsis_thermali:0.26669164764789116129,(344_Calothrix_sp._PCC_7507:0.19738579576554934891,(343_Chlorogloeopsis_fritschii:0.03367535000960011904,(348_Fischerella_thermalis:0.00750919026819845195,(347_Fischerella_sp._JSC-11:0.00752208955470831143,351_Fischerella_muscicola:0.01509551153609045744):0.00377367191598056064[48]):0.00481111316449381841[41]):0.27458319613593307196[100]):0.12369131697863397501[91]):0.11234746793123563668[75]):0.09990052396329274220[56]):0.34493014412852696093[91]):0.08868454679730021495[40],((311_Halothece_sp._PCC_7418:0.36184912543596609025,((354_Stanieria_cyanosphaera_PCC:0.13861683323513052102,365_Cyanobacterium_aponinum_PC:0.41745089275444108301):0.15279104458386782994[89],((((313_Synechococcus_sp._PCC_7336:0.36264899763829450130,(345_Acaryochloris_sp._HICR111A:0.11332668813353313775,(346_Acaryochloris_marina_MBIC1:0.03163283262736107937,340_Acaryochloris_sp._CCMEE_54:0.02850773115773466010):0.09644550897983317217[100]):0.35233068399548295169[97]):0.07469760726518348548[35],317_Oscillatoria_sp._PCC_10802:0.40397070267246765196):0.02793605512141931688[2],((336_Leptolyngbya_sp._Heron_Isl:0.40472353753572970492,(316_Pleurocapsa_sp._PCC_7319:0.25705697491355716799,(358_Chroococcidiopsis_thermali:0.29743769201894865173,((327_Anabaena_variabilis_ATCC_2:0.13266004107897780284,331_Anabaena_sp._PCC_7108:0.21735975974557253387):0.08386426252305249907[94],((362_cyanobacterium_PCC_7702:0.11725562731331347355,353_Chlorogloeopsis_fritschii:0.08647027731636243442):0.07666840461014259867[96],((342_Fischerella_sp._JSC-11:0.05157647863080040002,352_Fischerella_muscicola:0.01514658041891360669):0.01970698132001011404[70],(350_Fischerella_muscicola:0.02864901331145901578,349_Fischerella_sp._PCC_9339:0.05284123052499836021):0.01561271041402907331[88]):0.02227182118365330851[57]):0.07838181991148961136[87]):0.11395683030842175543[85]):0.12818373365152918586[89]):0.03685699001422012427[22]):0.06678898689262312283[54],(((359_Synechococcus_sp._JA-2-3B:0.08661015939453524382,361_Synechococcus_sp._JA-3-3Ab:0.04006888296330200772):0.37750971731894195083[100],((360_Scytonema_hofmanni:0.10018395659081956450,357_Microchaete_sp._PCC_7126:0.10151440333604525024):0.28847851005294267157[100],374_Acaryochloris_sp._CCMEE_54:0.56077817253470674252):0.18313016244680568878[77]):0.02733923552104487037[12],((320_Leptolyngbya_sp._Heron_Isl:0.02906055632888334198,329_Leptolyngbya_sp._PCC_7375:0.07934867651670861333):0.16089001839151112483[100],337_Synechococcus_sp._PCC_7335:0.25903422789693097972):0.20869783205411288063[95]):0.03147790099513237544[2]):0.01520125868672031130[1]):0.04523276700010441659[2],(334_Pleurocapsa_sp._PCC_7327:0.15942940872492455950,((330_Cyanothece_sp._PCC_7822:0.17672117190926131536,338_Cyanothece_sp._PCC_7424:0.12909287833304289306):0.16068240762411029054[90],((319_Cyanothece_sp._PCC_8802:0.01122132396714871361,318_Cyanothece_sp._PCC_8801:0.01843355786763126863):0.16365503358312102899[98],((328_Crocosphaera_watsonii_WH_0:0.00000100000050002909,(332_Crocosphaera_watsonii_WH_8:0.00555364310534134896,333_Crocosphaera_watsonii_WH_8:0.00000100000050002909):0.00561820538788156566[59]):0.15721923948353810907[94],321_Cyanothece_sp._ATCC_51142:0.13184866647020795916):0.15059856528228615646[92]):0.06125688838996597857[47]):0.07807740667931131751[63]):0.15751327708395024274[68]):0.02375461838540999723[6]):0.16688155830644613165[24]):0.08450469360102830230[19],(((((272_Synechococcus_sp._RS9917:0.14103412591835221002,(285_Cyanobium_sp._PCC_7001:0.13264440690088119545,290_Synechococcus_sp._CB0101:0.11053704457022618668):0.04509726294432059301[42]):0.08044412879508212877[76],(325_Synechococcus_sp._CB0101:0.35224323456219569506,323_Synechococcus_sp._CB0205:0.36881310556876195639):0.31135476754192703552[100]):0.08241258891364143524[77],(((230_Xenococcus_sp._PCC_7305:0.19578790827527373097,(201_Anabaena_sp._PCC_7108:0.07688979977000361121,143_Anabaena_cylindrica_PCC_71:0.10176989863465618624):0.07284074425045386492[81]):0.03739168516051830787[58],(((200_Arthrospira_platensis_str.:0.00589698274127272923,(197_Arthrospira_sp._PCC_8005:0.00000100000050002909,199_Arthrospira_platensis_C1:0.00486165449790294888):0.00447413206889435595[72]):0.14357690292361921514[100],(190_Synechococcus_sp._PCC_7002:0.03434134594064516127,283_Leptolyngbya_sp._PCC_7376:0.22385182369643608080):0.07647880591285630136[89]):0.06721986445004510258[38],((247_Synechocystis_sp._PCC_6803:0.01508303079611037628,228_Synechocystis_sp._PCC_6803:0.00000100000050002909):0.18641034986351440184[100],(277_Geminocystis_herdmanii:0.08426825889879675791,270_Cyanobacterium_aponinum_PC:0.07067469255239981762):0.07963886523780124016[99]):0.03963190583905008835[64]):0.01170306768200958872[16]):0.02718700766448586131[15],269_Halothece_sp._PCC_7418:0.16480831540475579522):0.01252938333222282548[8]):0.06104166923400801414[66],(((301_Fischerella_muscicola:0.08207637376865206669,(312_Calothrix_sp._PCC_7103:0.13061820675169580341,(297_Nostoc_sp._PCC_7524:0.04478425884141134378,(299_Nostoc_sp._PCC_7120:0.00000100000050002909,310_Anabaena_variabilis_ATCC_2:0.00466432098487372122):0.02316998556222376465[88]):0.03198297493799705238[94]):0.02545002769336922668[71]):0.08007760922623261002[88],((243_Pseudanabaena_sp._PCC_6802:0.14250706304043270212,(282_Nodosilinea_nodulosa:0.15876046022152698334,298_Cyanothece_sp._PCC_7425:0.25041899877353068771):0.02611272760664684958[41]):0.05472756898560102168[69],((263_Leptolyngbya_sp._Heron_Isl:0.05335285849885339909,287_Leptolyngbya_sp._PCC_7375:0.07412772235628571627):0.15057516423297639951[100],(251_Leptolyngbya_boryana:0.10788775066165487426,308_Cyanothece_sp._PCC_7424:0.19930845966633456423):0.04581332415610003123[21]):0.01428596578629015221[11]):0.01348967248830620740[5]):0.03613161670648427221[39],(242_Microcoleus_sp._PCC_7113:0.12892528784459611479,((275_Oscillatoria_sp._PCC_10802:0.10321354849199347592,(264_Oscillatoria_sp._PCC_6506:0.07897476587424015482,286_Oscillatoria_acuminata_PCC:0.13537823071252944040):0.02948295634190011005[44]):0.06026057439263680926[93],(249_Geitlerinema_sp._PCC_7105:0.14380141945166796669,(296_Cyanothece_sp._ATCC_51142:0.03168511813965103896,293_Cyanothece_sp._CCY0110:0.02122028534181531062):0.12540998720050766657[98]):0.05978457773882736581[62]):0.03327394285681090774[30]):0.06668582245427842614[45]):0.07479287900096995290[84]):0.01554912509183738537[9],(((164_Nostoc_punctiforme_PCC_731:0.08072888792209563513,219_Nostoc_sp._PCC_7524:0.11710105294659546582):0.06905372492450916055[88],(((082_Chlorogloeopsis_fritschii:0.04311561359775689661,(145_Fischerella_muscicola:0.05780758928674566838,(109_Fischerella_sp._JSC-11:0.01059004368059097805,124_Fischerella_muscicola:0.00504619864601014902):0.02427001318767855326[94]):0.05173676475220002902[94]):0.00998826683286197815[49],(077_cyanobacterium_PCC_7702:0.00000100000050002909,137_Mastigocladus_laminosus:0.01021753783054104615):0.06032042365272469175[100]):0.04643065027178627097[91],119_Gloeocapsa_sp._PCC_7428:0.12746490562442541750):0.01482700472382532213[32]):0.06734422863025796291[88],(((((108_Synechococcus_elongatus_PC:0.06635622652795479526,(((((((210_Synechococcus_sp._RS9917:0.01375953460102143609,(212_Synechococcus_sp._WH_7805:0.02001380950276255774,213_Synechococcus_sp._WH_7803:0.02797436953396758089):0.00828474505092971665[65]):0.00861890817633468448[52],(((208_Synechococcus_sp._WH_8109:0.01860248413253318481,226_Synechococcus_sp._WH_8102:0.03782828885890703735):0.01725258422911088441[78],(236_Synechococcus_sp._BL107:0.00543405819845598978,238_Synechococcus_sp._CC9902:0.01899029086492834070):0.04615119254115519071[99]):0.02162054638346471228[49],(233_Synechococcus_sp._WH_8016:0.03301306950183607869,239_Synechococcus_sp._CC9311:0.04673939420878942680):0.03140615944717371766[94]):0.02509196664468567664[45]):0.00990733618438957228[10],214_Synechococcus_sp._RS9916:0.03142045874251723353):0.02403204737743354358[26],206_Cyanobium_gracile_PCC_6307:0.07123352521106315893):0.01389344747632254191[11],(((215_Prochlorococcus_marinus_st:0.00000100000050002909,216_Prochlorococcus_marinus_st:0.00495693559776778318):0.06761925649489763934[100],(241_Prochlorococcus_marinus_st:0.00016534061769303324,(((258_Prochlorococcus_marinus_st:0.00482225226256625429,((253_Prochlorococcus_marinus_st:0.00000100000050002909,315_Prochlorococcus_marinus_st:0.00000100000050002909):0.00477673677133248591[66],257_Prochlorococcus_marinus_st:0.00000100000050002909):0.00479388505905748233[77]):0.00464307561486113768[78],((271_Prochlorococcus_sp._W7:0.00485164171857040225,(274_Prochlorococcus_sp._W4:0.00000100000050002909,(278_Prochlorococcus_sp._W8:0.00000100000050002909,267_Prochlorococcus:0.00000100000050002909):0.00000100000050002909[94]):0.00000100000050002909[74]):0.00000100000050002909[97],266_Prochlorococcus_marinus_st:0.06555736801362536947):0.01082488443041694504[59]):0.05559189598128598531[75],(240_Prochlorococcus_marinus_su:0.00000100000050002909,246_Prochlorococcus_marinus:0.00491831310607898035):0.00923303989676916778[96]):0.02775068202834953326[43]):0.15351532972124268261[100]):0.10170507567570515051[92],(224_Synechococcus_sp._RCC307:0.08384039000408574638,187_Cyanobium_sp._PCC_7001:0.05939734470769113261):0.00802921542801207644[28]):0.01014064455763813259[42]):0.03692715559638366785[35],176_Synechococcus_sp._WH_5701:0.08903167568904461759):0.02331509724921490903[43],(194_Synechococcus_sp._CB0101:0.03975198884891870121,169_Synechococcus_sp._CB0205:0.05829018482635355686):0.02082304805479124818[51]):0.08832701764304703995[94]):0.05337953720490601350[36],(088_Geitlerinema_sp._PCC_7407:0.08183839380706411926,017_Oscillatoriales_cyanobacte:0.09275736149862356950):0.03839920911188358932[55]):0.02050729341842049899[6],(117_Pleurocapsa_sp._PCC_7327:0.11704611586178981231,((255_Oscillatoriales_cyanobacte:0.17141070099857089182,((295_Calothrix_sp._PCC_7507:0.04002278279612557499,(300_Chroococcidiopsis_thermali:0.10958891221042539599,(305_Fischerella_sp._JSC-11:0.00491890846270687330,(303_Chlorogloeopsis_fritschii:0.00000100000050002909,306_Fischerella_muscicola:0.00483057777594538516):0.00000100000050002909[44]):0.09808325605014420190[100]):0.03086427946896028521[77]):0.03375492475195409486[81],(326_Synechococcus_sp._PCC_7335:0.20612959252215876105,254_Pleurocapsa_sp._PCC_7327:0.18419677448837559752):0.04528385732135378067[39]):0.08019507011432515375[62]):0.18563972516540935831[100],(165_Acaryochloris_marina_MBIC1:0.00360783258699805915,155_Acaryochloris_sp._HICR111A:0.00619886768286643207):0.16573979301265712172[100]):0.05967230395519336483[47]):0.02781248548197018042[19]):0.02218739008150631339[0],(((178_Prochlorothrix_hollandica:0.05242619191039397941,(209_Prochlorothrix_hollandica:0.00000100000050002909,191_Prochlorothrix_hollandica:0.01337871048509123827):0.00917356736642621108[92]):0.13262490151117853343[100],149_Nodosilinea_nodulosa:0.07446641544517362821):0.06250807871093523593[37],144_Moorea_producens_3L:0.06298029244674123450):0.02901990999622464096[8]):0.02395694307831709377[0],(((((160_Dactylococcopsis_salina_PC:0.00463444398627189513,159_Dactylococcopsis_salina_PC:0.00000100000050002909):0.02910122038252119236[100],151_Halothece_sp._PCC_7418:0.00394227083737010413):0.07348522776563200243[87],076_Coleofasciculus_chthonopla:0.05125388611032485192):0.01119524993653651587[11],((096_Microcoleus_sp._PCC_7113:0.02288236487245622411,070_Microcoleus_sp._PCC_7113:0.00000100000050002909):0.04226338092811115360[99],(025_Microcoleus_sp._PCC_7113:0.05931307393385180926,(((166_Gloeocapsa_sp._PCC_73106:0.10334715269167414620,((167_Leptolyngbya_sp._Heron_Isl:0.00592014478776794594,172_Leptolyngbya_sp._PCC_7375:0.02971358957644095258):0.19038381837458145940[100],(128_Leptolyngbya_sp._PCC_7376:0.10263920028820440467,(((093_Leptolyngbya_sp._PCC_7376:0.03982881748847400677,048_Synechococcus_sp._PCC_7002:0.00000100000050002909):0.00492339893844205220[54],037_Synechococcus_sp._PCC_7002:0.00000100000050002909):0.00000100000050002909[45],161_Synechococcus_sp._PCC_7002:0.00558727549314280703):0.02589448984257594968[75]):0.01879758316885886058[56]):0.00000100000050002909[1]):0.02063179866908029444[1],((((153_Cyanothece_sp._PCC_7424:0.00000100000050002909,((162_Cyanothece_sp._PCC_7822:0.00991768231661270712,158_Cyanothece_sp._PCC_7822:0.00504515480709102904):0.01005739632555908113[61],154_Cyanothece_sp._PCC_7424:0.00501934271084069229):0.00000100000050002909[14]):0.05210808210119473316[98],(091_Pleurocapsa_sp._PCC_7327:0.00478375795020442689,083_Pleurocapsa_sp._PCC_7327:0.00000100000050002909):0.02854572469371385868[98]):0.00741957137769371639[17],((100_Synechocystis_sp._PCC_6714:0.00989008342816155746,075_Synechocystis_sp._PCC_6803:0.00480535290035142854):0.02989005618468151479[100],((307_Microcystis_aeruginosa_TAI:0.20011589439675420143,(((126_Microcystis_aeruginosa:0.00489441987866711618,138_Microcystis_aeruginosa_SPC:0.01028965180820233700):0.00000100000050002909[29],(114_Microcystis_aeruginosa_NIE:0.00489254595731966526,115_Microcystis_aeruginosa_PCC:0.00000100000050002909):0.00491944989217629226[71]):0.00000100000050002909[15],112_Microcystis_aeruginosa_SPC:0.00000100000050002909):0.00825693443172551694[87]):0.01249752245527851156[92],(((211_Geminocystis_herdmanii:0.02394763016728426730,(237_Cyanobacterium_aponinum_PC:0.02130256794879602233,182_Cyanobacterium_stanieri_PC:0.01019343785241493515):0.00683045160872609077[73]):0.04417968240254607076[94],(175_Cyanothece_sp._PCC_8802:0.01393980002990612317,(174_Crocosphaera_watsonii_WH_0:0.00000100000050002909,(((177_Cyanothece_sp._ATCC_51142:0.00000100000050002909,180_Cyanothece_sp._ATCC_51142:0.00505346748208341124):0.00507005848506730111[77],(324_Crocosphaera_watsonii_WH_0:0.00689035563699328703,173_Crocosphaera_watsonii_WH_8:0.00507979067664778450):0.01009265574894668664[33]):0.00100330016376211362[8],322_Crocosphaera_watsonii_WH_0:0.00000100000050002909):0.00402305382857566594[7]):0.01124560855056721577[50]):0.02082615680307409872[65]):0.02626775538081952896[54],(232_Cyanothece_sp._ATCC_51142:0.01695631609225491457,(207_Cyanothece_sp._PCC_8802:0.03500077906882092388,335_Cyanothece_sp._CCY0110:0.02079827252122577394):0.00702628689351880633[56]):0.07147079234529420122[100]):0.01698625731052492982[11]):0.01761297577178795246[16]):0.02013416137402622083[28]):0.00899527989364790580[10],((134_Xenococcus_sp._PCC_7305:0.10353399865029906668,(104_Stanieria_cyanosphaera_PCC:0.06992174734993601704,135_Pleurocapsa_sp._PCC_7319:0.10302695785460005973):0.02372956995851368753[52]):0.03525495300451340214[43],(((116_Pleurocapsa_sp._PCC_7319:0.01948839811273190073,095_Pleurocapsa_sp._PCC_7319:0.00482034377506261168):0.01029998505604237669[86],196_Pleurocapsa_sp._PCC_7319:0.01472597429385853231):0.03054118318941268992[99],(106_Stanieria_cyanosphaera_PCC:0.00502949445923395102,125_Stanieria_cyanosphaera_PCC:0.01932512203364400438):0.02978359121692911865[96]):0.02333697881015904183[83]):0.02272376672230996125[18]):0.00339880061378737596[4]):0.02283852726003724951[0],((085_filamentous_cyanobacterium:0.00000100000050002909,056_filamentous_cyanobacterium:0.00000100000050002909):0.03125123567873554914[100],023_Spirulina_subsalsa:0.03660998252658892449):0.03348904602627125054[53]):0.02820154819322519615[0]):0.03420013256095064819[0]):0.00998084840347273315[0]):0.00746110641804253408[0],((((260_Cyanobium_gracile_PCC_6307:0.04005483692381702626,220_Synechococcus_sp._WH_5701:0.02589943438658260169):0.00550041816234986877[45],(((((((261_Synechococcus_sp._CC9311:0.00000100000050002909,248_Synechococcus_sp._CC9311:0.00000100000050002909):0.00544800411449985512[91],256_Synechococcus_sp._WH_8016:0.00000100000050002909):0.00560416554329297607[94],(250_Synechococcus_sp._RCC307:0.04432271309637640655,(265_Synechococcus_sp._BL107:0.00000100000050002909,(268_Synechococcus_sp._WH_8109:0.00000100000050002909,(273_Synechococcus_sp._WH_8102:0.00000100000050002909,279_Synechococcus_sp._WH_8102:0.00482285811256049409):0.00481841437115282552[68]):0.01453114020077190226[89]):0.02942068509111688684[94]):0.00555150361801657992[66]):0.04006842876351635052[89],(227_Synechococcus_sp._RS9917:0.00000100000050002909,(231_Synechococcus_sp._WH_7803:0.00489760646632195727,229_Synechococcus_sp._WH_7803:0.00000100000050002909):0.00483093670149623965[59]):0.00524896747534930701[72]):0.00418168303406491696[57],225_Synechococcus_sp._RS9916:0.00131671509953212862):0.04034691146699420639[90],222_Cyanobium_sp._PCC_7001:0.00597779058704119275):0.01578854110248150561[46],003_Paulinella_sp._HSY-2012:0.02067171379875137535):0.01792603822613196909[32]):0.09659861780198944481[75],(046_Synechococcus_elongatus_PC:0.00483905134501867208,010_Synechococcus_elongatus_PC:0.00000100000050002909):0.01836763017573682030[100]):0.02402000597101679874[21],((028_Geitlerinema_sp._PCC_7407:0.00000100000050002909,009_Geitlerinema_sp._PCC_7407:0.00000100000050002909):0.06411797497697090120[100],((AuroraVandensisGreen:0.16144789476654625160,(195_Gloeobacter_kilaueensis_JS:0.00000100000050002909,(284_Gloeobacter_violaceus_PCC_:0.04493789016987301960,((288_Gloeobacter_violaceus_PCC_:0.05862301082251325512,292_Gloeobacter_kilaueensis_JS:0.06316164537304662374):0.09166461230556863238[99],(304_Gloeobacter_kilaueensis_JS:0.19747383782799660112,294_Gloeobacter_violaceus_PCC_:0.14487580487224935299):0.12755467979425211267[99]):0.00494197618701472819[26]):0.03020894335033571729[32]):0.07172620105963352366[90]):0.18326301287279958552[100],((((((((204_Leptolyngbya_sp._PCC_7375:0.02029162990016012205,(092_Leptolyngbya_sp._Heron_Isl:0.00568974553146032146,(146_Leptolyngbya_sp._PCC_7375:0.01366674402755725486,217_Leptolyngbya_sp._Heron_Isl:0.01047838735601888498):0.12427179588874505411[100]):0.01903649813551401213[16]):0.01134803728777558467[8],089_Leptolyngbya_sp._Heron_Isl:0.00738263630077451959):0.03333406543126108540[26],((073_Synechococcus_sp._PCC_7335:0.00000100000050002909,189_Synechococcus_sp._PCC_7335:0.00551553471311397748):0.01973681309717942658[97],(259_Nodosilinea_nodulosa:0.00912319517535091322,(262_Nodosilinea_nodulosa:0.00521169095747574859,244_Nodosilinea_nodulosa:0.00000100000050002909):0.01197588094004771453[95]):0.05469885320964918557[95]):0.00941258017408170070[30]):0.04452133955018218459[16],(156_Geitlerinema_sp._PCC_7105:0.06453584420075030026,170_Oscillatoria_acuminata_PCC:0.05736644118754009175):0.00522259948777669975[6]):0.01197100431357719756[0],((007_Oscillatoria_formosa:0.02990737641790083140,(020_Microcoleus_vaginatus_FGP-:0.00484796884341100621,((((((014_Oscillatoria_nigro-viridis:0.00947405842670857867,026_Oscillatoria_nigro-viridis:0.00468459151532842500):0.00000100000050002909[18],019_Oscillatoria_nigro-viridis:0.00000100000050002909):0.00000100000050002909[19],016_Oscillatoria_nigro-viridis:0.00962202460047750785):0.00466488183178573237[13],021_Oscillatoria_nigro-viridis:0.00000100000050002909):0.01430430047917589931[64],(008_Oscillatoria_nigro-viridis:0.02396365637018391462,011_Microcoleus_vaginatus_FGP-:0.00483237454216154522):0.02417510255707525785[94]):0.00000100000050002909[16],006_Microcoleus_vaginatus_FGP-:0.00000100000050002909):0.00464383110732774929[17]):0.01368897221544820332[72]):0.00503312032238060862[41],((185_Trichodesmium_erythraeum_I:0.00509140600632132165,186_Trichodesmium_erythraeum_I:0.00497159219931002372):0.02696963728063674764[100],((130_Oscillatoria_sp._PCC_10802:0.00000100000050002909,(131_Oscillatoria_sp._PCC_10802:0.00000100000050002909,(132_Oscillatoria_sp._PCC_10802:0.00519774808138901041,133_Oscillatoria_sp._PCC_10802:0.00520727430170655342):0.00520906589087123673[35]):0.01043248618419227551[43]):0.02436631525567986567[50],(((252_Lyngbya_sp._PCC_8106:0.00000100000050002909,309_Lyngbya_sp._PCC_8106:0.00000100000050002909):0.07984501575652112881[100],(202_Arthrospira_platensis_NIES:0.00000100000050002909,205_Arthrospira_platensis_C1:0.00955441818082217696):0.03105525043551013442[100]):0.02838699176716290296[66],(024_Lyngbya_aestuarii_BL_J:0.00000100000050002909,045_Lyngbya_sp._PCC_8106:0.00966556801818661653):0.08604604567016103933[100]):0.01296379087407505473[43]):0.01854093609473420404[20]):0.02866600441667459601[23]):0.02191728047129457707[8]):0.01650030285810347164[0],((((((276_Synechococcus_sp._PCC_7336:0.03782367264601173285,235_Synechococcus_sp._PCC_7336:0.20994859167435464542):0.11149621742600587804[99],(188_Synechococcus_sp._JA-2-3B:0.00000100000050002909,(181_Synechococcus_sp._JA-3-3Ab:0.00000100000050002909,183_Synechococcus_sp._JA-3-3Ab:0.00511469261117373543):0.00511742894049101432[32]):0.10116038761580464778[98]):0.06565477079178767827[67],((((163_Pseudanabaena_sp._PCC_6802:0.06673265705591284458,(140_Pseudanabaena_sp._PCC_6802:0.00961067700439835471,136_Pseudanabaena_sp._PCC_6802:0.00000100000050002909):0.01763545283505421959[100]):0.03200577581330250887[71],094_Pseudanabaena_sp._PCC_7367:0.06910738617999442890):0.00876055436239631113[27],198_Pseudanabaena_biceps_PCC_7:0.04976501562187393007):0.02292142187006850768[36],072_Synechococcus_sp._PCC_7502:0.04845690596831305624):0.07551279154142685746[97]):0.04602118001469386599[8],((147_Cyanothece_sp._PCC_7425:0.06410750147239793750,121_Cyanothece_sp._PCC_7425:0.00246643348080453342):0.07501806223870040313[100],((152_Thermosynechococcus_sp._NK:0.02196829459055676947,142_Thermosynechococcus_elonga:0.00701621564153985609):0.10837752409867158765[100],(((127_Thermosynechococcus_sp._NK:0.00733726825304206916,(129_Thermosynechococcus_vulcan:0.00933531015715324472,(110_Synechococcus_elongatus:0.00466144536227651972,102_Thermosynechococcus_elonga:0.00000100000050002909):0.00000100000050002909[31]):0.01163277034246912485[94]):0.01717922069655700504[91],141_Synechococcus_sp._PCC_6312:0.08002616589938510439):0.04558937035262507903[56],(118_Synechococcus_sp._PCC_6312:0.05683101366638602242,(063_Thermosynechococcus_sp._NK:0.00010514548369247725,078_Thermosynechococcus_elonga:0.00940197538089103738):0.02647269809618653727[81]):0.02907427589636803894[40]):0.01993764954233277417[23]):0.05841426609063908593[67]):0.01982470924076826235[28]):0.01419792915861129746[0],((((105_Microchaete_sp._PCC_7126:0.05789710038228033928,139_Calothrix_sp._PCC_7507:0.05190864006006671444):0.02823099622897148192[25],(066_Nostoc_sp._PCC_7524:0.01654146344476426986,(051_Nostoc_sp._PCC_7107:0.01062156022705155502,(057_Anabaena_variabilis_ATCC_2:0.00946810807862999600,068_Nostoc_sp._PCC_7120:0.00000100000050002909):0.01798715042136949868[79]):0.01149554844948855181[44]):0.01487229614694500317[70]):0.01689142443531175750[15],(((((((035_Anabaena_sp._PCC_7108:0.00000100000050002909,003_Anabaena_sp._PCC_7108:0.00000100000050002909):0.00570912519499467124[83],042_Anabaena_sp._PCC_7108:0.00478892481651621051):0.00913047065417617841[61],((031_Anabaena_cylindrica_PCC_71:0.00000100000050002909,(((058_Microchaete_diplosiphon:0.02945961585916026526,((101_Calothrix_sp._PCC_7507:0.00627586762916703539,(123_Microchaete_sp._PCC_7126:0.00476471464481380454,120_Microchaete_sp._PCC_7126:0.00000100000050002909):0.02302178135468064407[100]):0.02832867249637024271[93],038_Nostoc_punctiforme_PCC_731:0.03979622109810555114):0.01053695558444146807[38]):0.01510614036430906609[12],(((049_Nostoc_sp._PCC_7120:0.00000100000050002909,044_Anabaena_variabilis_ATCC_2:0.00953349469495373171):0.03041456538457040434[96],(013_Nostoc_sp._PCC_7524:0.01946840918698529085,022_Nostoc_sp._PCC_7107:0.02972450838128927117):0.00956579158601203634[32]):0.01451397375803376022[19],(074_Cylindrospermopsis_racibor:0.00482009987475805549,055_Raphidiopsis_brookii_D9:0.00967076911177377693):0.01490364307086465209[97]):0.00000100000050002909[6]):0.01432179958113658641[3],(((005_Nodularia_spumigena:0.00000100000050002909,150_Nodularia_spumigena_CCY941:0.00000100000050002909):0.02078388616887760776[98],(027_Nodularia_spumigena_CCY941:0.00000100000050002909,012_Nodularia_spumigena_CCY941:0.01804367584356741938):0.00095001587116591525[10]):0.05248176990744003040[94],(001_Cylindrospermum_stagnale_P:0.00000100000050002909,067_Cylindrospermum_stagnale_P:0.00480040723307876595):0.05437980032336519470[84]):0.00000100000050002909[2]):0.01666584931964089702[8]):0.03631572793516644332[18],113_Anabaena_sp._90:0.06130181949343472408):0.01911546031489725489[20]):0.01452770074568599142[6],((062_Nostoc_azollae_0708:0.00941678320346507093,065_Nostoc_azollae_0708:0.01438044405720012228):0.01049423586376245325[56],(029_Anabaena_cylindrica_PCC_71:0.00478535557199642227,032_Anabaena_cylindrica_PCC_71:0.00000100000050002909):0.00510597378457101209[77]):0.02444824848877072757[82]):0.01804833381317301130[9],(079_Cylindrospermopsis_racibor:0.00000100000050002909,((289_Cylindrospermopsis_racibor:0.02509335037232059487,084_Raphidiopsis_brookii_D9:0.00463719928261310116):0.00012049311829480856[47],080_Cylindrospermopsis_racibor:0.00000100000050002909):0.00477043187878036044[52]):0.02020608331193437385[95]):0.01430374191502560045[19],((103_Cylindrospermum_stagnale_P:0.01866292621337784993,099_Cylindrospermum_stagnale_P:0.01087737796861627804):0.01040005040805680459[84],086_Anabaena_sp._90:0.06358878820586473524):0.02843289979109040952[26]):0.00000100000050002909[6],122_Nostoc_punctiforme_PCC_731:0.09836908962286455838):0.02977414740708086635[13]):0.05655702078423649470[29],(((((192_cyanobacterium_PCC_7702:0.05821900638905062875,(087_Chlorogloeopsis_fritschii:0.00464780738725797601,061_Chlorogloeopsis_fritschii:0.00000100000050002909):0.00000100000050002909[97]):0.02974272703378559243[88],(052_Fischerella_sp._JSC-11:0.03702549388600289565,(245_Fischerella_muscicola:0.00000100000050002909,(281_Fischerella_sp._PCC_9339:0.00000100000050002909,280_Fischerella_sp._PCC_9339:0.00000100000050002909):0.01841470471921163171[96]):0.03592473427179138090[94]):0.00923806570296759576[19]):0.00623227257182760314[24],((060_Fischerella_muscicola:0.00965006143728446703,157_Fischerella_sp._PCC_9339:0.00942196276539876941):0.05866538970018628923[98],((004_Fischerella_sp._JSC-11:0.03946023485279035597,(314_Chlorogloeopsis_fritschii:0.02031789562716942651,168_cyanobacterium_PCC_7702:0.01854983886919567629):0.00651703644161334954[54]):0.01168514649588852858[35],179_Chlorogloeopsis_fritschii:0.11261858341698680053):0.01043114730176378235[40]):0.02013130324319645573[22]):0.01700263528718554476[6],((030_Calothrix_sp._PCC_6303:0.02400068746774017270,(041_Calothrix_sp._PCC_7103:0.00000100000050002909,(039_Calothrix_sp._PCC_7103:0.00000100000050002909,(034_Calothrix_sp._PCC_7103:0.00000100000050002909,040_Calothrix_sp._PCC_7103:0.00478095751273972195):0.00952877496346646660[88]):0.00475503239249879753[65]):0.01988754772460910730[88]):0.02381778251100579585[73],(((193_Chroococcidiopsis_thermali:0.04837425177118191005,(018_Gloeocapsa_sp._PCC_7428:0.01489562180137738757,(171_Gloeocapsa_sp._PCC_7428:0.05019507882255286324,(218_Leptolyngbya_sp._Heron_Isl:0.00917042397897849942,203_Leptolyngbya_sp._PCC_7375:0.00000100000050002909):0.17417153773160778951[100]):0.08504426860520966636[82]):0.03038942801469476929[14]):0.03610029396889658654[18],((((047_Scytonema_hofmanni:0.02505920678007571636,(054_Chroococcidiopsis_thermali:0.09098973252776923482,184_Calothrix_sp._PCC_7103:0.04466479518166271978):0.03780978605418880423[66]):0.02157572375323902475[24],(111_Rivularia_sp._PCC_7116:0.11551739569361826887,033_Mastigocladopsis_repens:0.02977206610855811716):0.00568642093948860602[13]):0.02689093823358372840[5],043_Mastigocladopsis_repens:0.02452629049601416991):0.00598016406348652208[0],(098_Rivularia_sp._PCC_7116:0.06683136317309558427,(081_Scytonema_hofmanni:0.00000100000050002909,(090_Scytonema_hofmanni:0.00000100000050002909,(064_Scytonema_hofmanni:0.01435565807247518415,097_Scytonema_hofmanni:0.00000100000050002909):0.00478545863523808634[34]):0.00967588081508238829[51]):0.03931640358036495031[94]):0.01588656196633507836[21]):0.01019517519365016958[6]):0.01042875123848486033[0],(015_Richelia_intracellularis_H:0.00000100000050002909,302_Richelia_intracellularis_H:0.00627941414695609799):0.06637912512894707240[100]):0.00560432325929943482[0]):0.01081133331010617968[0]):0.01026354194342066900[0],(((008_Galdieria_sulphuraria:0.12170013313919492148,003_Cyanidioschyzon_merolae_st:0.12204994672331545558):0.04651411154651381025[24],(((006_Heterosigma_akashiwo:0.02860563737481485250,007_Vaucheria_litorea:0.04699627617523141621):0.02139921868138398853[84],009_Chaetoceros_simplex:0.05105476104672254656):0.06970320980450216142[82],((007_Cyanophora_paradoxa:0.12851490260854173120,((008_Lepidodinium_chlorophorum:0.06875899417174208272,(006_Chlorella_sp._ArM0029B:0.04410192596533391712,(005_Eutreptia_viridis:0.07466681320317303250,(011_Euglena_stellata:0.06725177951740042570,004_Eutreptiella_gymnastica:0.01726374207161980590):0.00633204373812208329[42]):0.01840609400343777222[68]):0.01979896475292278885[62]):0.03233186325135824907[78],(002_Sphagnum_portoricense:0.01545393886900332803,(004_Hamamelis_mollis:0.00000100000050002909,005_Helianthus_maximiliani:0.00496887990230656350):0.03108401300139365317[99]):0.02079470024753881513[80]):0.03370731740098552309[64]):0.02239959366859453974[34],(001_Porphyridium_purpureum:0.04925159442689801270,(002_Guillardia_theta:0.01012284397411256087,001_Rhodomonas_salina:0.01418014245336939386):0.08798100315219585521[100]):0.01924018023609517722[39]):0.00697927280638472626[2]):0.01647749014450549179[16]):0.01680327725015200516[13],(012_Karlodinium_veneficum:0.27378819408333643359,010_Emiliania_huxleyi:0.01869730808266992575):0.13745468167826135897[99]):0.10123421764494892894[92]):0.00566996861963366128[0]):0.00818899905735407350[0]):0.01842567724542052907[0],((069_Chamaesiphon_minutus_PCC_6:0.00132070070010574872,(002_Chamaesiphon_minutus_PCC_6:0.00000100000050002909,107_Chamaesiphon_minutus_PCC_6:0.00951779496715756020):0.04222140758440703218[100]):0.09658762660378869547[99],(148_Calothrix_sp._PCC_6303:0.14089772997375960006,050_Crinalium_epipsammum_PCC_9:0.01359740290552761098):0.03733545832584755225[51]):0.01550087322826238651[6]):0.02495400099178494979[0]):0.00567810010269975598[0],(053_Leptolyngbya_boryana:0.04090863029646601284,071_Leptolyngbya_boryana:0.08337950984714012637):0.03578837546753720761[18]):0.01103708933401360143[0],(036_Oscillatoriales_cyanobacte:0.00000100000050002909,059_Oscillatoriales_cyanobacte:0.00977689324582451356)OROOT:0.06558262824734059637[100]):0.01571011889897774849[0]):0.00602897501555527387[0]):0.03070723755533793023[0]):0.00761226536509168432[0]):0.02776091599491702089[0]):0.03781763574793201022[7]):0.03301344023613635159[10]):0.15209448214117043952[69]):0.15455455108920906904[12]):2.48205562223016);
