# Supplementary material for: A phylogenetically novel cyanobacterium most closely related to Gloeobacter
Source: ISME J. 2020 May 18;14(8):2142–52. doi: 10.1038/s41396-020-0668-5 (PMC7368068; doi:10.1038/s41396-020-0668-5)
Supplement: Supplementary file 10 — Supplemental File 6. [file 41396_2020_668_MOESM10_ESM.docx]

(((S15B_MN24_RAAC_196:0.00000100000050002909,GCA_002083785.1_ASM208378v1_genomic:0.00000100000050002909):0.56582330216053156047[100],(HGWMelainabacteria1:0.31101982364002012105,GL2-53_LSPB_72:0.28842889354541201286):0.42011966262170530317[100]):0.0624842137171539[100],(((((RIFCSPLOWO2_02_FULL_35_15:0.00000100000050002909,RIFCSPLOWO2_12_FULL_35_11:0.00000100000050002909):0.63080727934622937703[100],2554235000:1.43714970287712362484):0.16519642328610364967[100],2556921048:0.57134358424608744365):0.11968034578113019573[100],(Vampirovibrio_chlorellavorus_isolate_ncimb:0.61730866092690450664,(((RIFOXYA12_FULL_32_12:0.00472929956209507552,GWF2_32_7:0.00532069762294201318):0.22566229761635656081[100],(GWA2_34_9:0.22007416949567365494,GWF2_37_15:0.23828662400356606654):0.08829184381703626683[100]):0.09735466259385883170[100],((((HUM_7:0.00000100000050002909,HUM_12:0.00091912232225586042):0.06166419954516699081[100],(Zag111:0.00000100000050002909,2531839741:0.00000100000050002909):0.07430838503846287635[100]):0.06931503087381676864[100],(((Melainabacteria_sp._35_41:0.00107859427540267548,MH37:0.00401243656683287087):0.00993215223226175982[100],(Zag1:0.00000100000050002909,2523533517:0.00000100000050002909):0.00481859898828940463[100]):0.09632506790458954249[100],2523533519:0.13991942750462826872):0.03105185200032070658[82]):0.04700995928087110809[99],((HUM_10:0.00110095056404935264,(HUM_16:0.00000100000050002909,(HUM_23:0.00000100000050002909,(HUM_19:0.00000100000050002909,HUM_17:0.00099155310584513262):0.00000100000050002909[38]):0.00100027114741869558[97]):0.00017915319590741687[84]):0.00432057164452628092[98],((HUM_3:0.00000100000050002909,(HUM_4:0.00000100000050002909,HUM_5:0.00000100000050002909):0.00090700282922068351[100]):0.00037127430031036690[86],(((HUM_8:0.17740640993501144429,HUM_11:0.00000100000050002909):0.00019442019569533583[60],HUM_13:0.00515328929178883021):0.00113207408970200977[81],(HUM_18:0.00622479006427861803,HUM_15:0.00179992929768503724):0.00325043319166180584[100]):0.00274109284826804748[62]):0.00530813956751027306[84]):0.14651177873164786258[100]):0.30023381214209732271[100]):0.17563981597151495651[100]):0.14795470511894023846[100]):0.13234478407872279915[100],(((AuroraVandensis_green:0.00018085007492952800,AuroraVandensis_purple:0.00000100000050002909):0.41973557543210476828[100],(Gloeobacter_violaceus_PCC_7421:0.10106376969267252819,GloeobacterkilaueensisJS1:0.09942998301054800647):0.21480740652337632079[100]):0.12344321091570076288[100],(((Gloeomargarita_lithophora:0.00000100000050002909,2718217921:0.00000100000050002909):0.37790291942709430817[100],2508501011:0.51701780843713307512):0.06182400988173899353[82],(2504643012:0.38269656292881343296,(((2757320732:0.17917930071618534682,(2509601026:0.17046267182617605429,2627853929:0.17742283401367064943):0.03177209117641457276[54]):0.08581458294509994311[100],2509276045:0.26585724377134067886):0.02871241801747675612[34],(2503538020:0.18617419246804484123,(((2786546530:0.16412998139514967111,(2802429464:0.11766278443163134815,2773857838:0.15548381443238870614):0.09010233787741449285[100]):0.01968997101592236468[58],2636415546:0.19991763375017804205):0.02166738037372400311[63],((((2509276028:0.15908473386696231344,2636416084:0.17992749665994456354):0.04982745554899015161[100],(2786546746:0.15156107700352164369,(2576861326:0.13938253522176416443,2687453185:0.15744134720655481030):0.03460029233625791512[97]):0.10492428998642396032[100]):0.03283213220747972783[87],(2744054668:0.36303175582437396951,(2751185667:0.13481565941025963618,2627853561:0.22790123124376771013):0.05112051003390032100[100]):0.01426337477067815898[18]):0.01542618144335454293[20],(((2504643013:0.12721142207693825199,(2802429465:0.17342677525056399146,2510436000:0.27446262984954561404):0.03394284938692591208[85]):0.03488248825593072422[100],(((2651869645:0.06101112193239348336,2788500227:0.11038488963835343948):0.07514903974804232334[100],(2788500226:0.13064471333615071402,2503754017:0.09434066393002100981):0.02154198052986240966[93]):0.02601429295835695071[100],((2627853647:0.08226645813134021712,(((2562617131:0.04037974991721671181,2713896943:0.05676324336124694697):0.04856821732009125592[100],2775506824:0.06801122048191107772):0.01076966769222769023[22],((((2788499917:0.07417460919601855107,2775506855:0.05473887060993089942):0.01769427633004902467[97],2503982047:0.04621147250740456625):0.02127115499725088649[100],2509601025:0.05529302110270691711):0.01794346988529656531[100],(2509601027:0.05994699583904374712,(2775506883:0.03099988099671440595,(2775506992:0.00709672937276374129,((2775506816:0.00000100000050002909,2775506875:0.00000100000050002909):0.00506816444561628172[100],2775506820:0.00659745960729839383):0.00323219117490145154[79]):0.02174406673231394499[100]):0.02700735909304545490[100]):0.01197873117983264471[93]):0.00925755952796817987[33]):0.01434536963186942155[61]):0.02171121510395579782[100],((((((2529292565:0.00472829163102149105,2767802765:0.00375270030390690646):0.01226106869861215941[100],2630968419:0.23901939536637017980):0.01813393791322286813[100],2687453106:0.02187365809604377778):0.03700541750577753669[100],2551306142:0.04772323424967419431):0.03194818449417535788[100],(2802429304:0.04144578418275104287,(2517093042:0.05018446703674916270,2758568003:0.03804834914322186706):0.00936914165618867555[44]):0.03742643430880646105[100]):0.01278912385224690099[77],((2724678989:0.11924335255909258857,2510065008:0.15490187323265455777):0.03658421626973799162[100],2579778779:0.19661349024674848462):0.03006480017007358285[100]):0.01307615695833338337[72]):0.05920065113532544526[100]):0.06862353938117358998[100]):0.01801963408706543437[72],((2825676585:0.14232593067902690742,2509276031:0.10490383323039023655):0.05286135465549660767[100],((((2795385392:0.11742890484479072410,(2509276056:0.08275824804925054068,2503538028:0.06732141116326538499):0.03276712152906981895[100]):0.16131937430779952347[100],2524023186:0.23789106924604400128):0.07495622168791020445[100],2506520014:0.25368450078276427728):0.02775917689933755234[67],((((2503707009:0.09479073085252363862,2675903523:0.09714708271871180800):0.21110372516624556494[100],(2503754019:0.11649465208210189882,((2597490276:0.12348146126252669552,2775506821:0.14023919577648669144):0.04040244464032830690[100],2508501034:0.16565153757460895911):0.03621269036666184549[98]):0.05652818503793479582[100]):0.03974950464222184315[97],(2509276061:0.13774083949991006159,((2786546931:0.15667466441505098484,2721755494:0.17392941944211937066):0.05083854697133333156[100],(2808606688:0.23334726127564944798,2528768021:0.28936065984875519730):0.03939139138050058025[96]):0.01984456248737996586[62]):0.03878738860010653294[100]):0.02029104991617797574[91],2510461037:0.21075883070648679385)OROOT:0.03090473449635756931[96]):0.07234642971505635123[100]):0.02414686967461839376[96]):0.02855385571270581749[100]):0.02240470085262371777[44]):0.02003775424154687068[27]):0.02641447694738802143[38]):0.04675988792080480738[74]):0.05537155513525008482[90]):0.12947469471580061451[100]):0.32578546118160067735[100]):0.0624842137171539);
